# Human alphacoronavirus replication and innate immune induction in airway culture systems

**DOI:** 10.1128/mbio.03203-25

**Published:** 2025-12-10

**Authors:** Alejandra Fausto, Clayton J. Otter, Leonel Torres, Ebba K. Blomqvist, Nicole Bracci, David M. Renner, Li Hui Tan, Devon Mooring, Nadine Ebert, Bettina Trüeb, Volker Thiel, Noam A. Cohen, James M. Burke, Susan R. Weiss

**Affiliations:** 1 Department of Microbiology, Perelman School of Medicine, University of Pennsylvania 332252, Philadelphia, Pennsylvania, USA; 2 Center for Research on Coronaviruses and Other Emerging Pathogens, Perelman School of Medicine, University of Pennsylvania 14640 , Philadelphia, Pennsylvania, USA; 3 Institute for Immunology & Immune Health, Perelman School of Medicine, University of Pennsylvania 730444, Philadelphia, Pennsylvania, USA; 4 Department of Molecular Medicine, The Herbert Wertheim University of Florida Scripps Institute for Biomedical Innovation and Technology, Jupiter, Florida, USA; 5 Department of Immunology and Microbiology, The Herbert Wertheim University of Florida Scripps Institute for Biomedical Innovation and Technology, Jupiter, Florida, USA; 6 Skaggs Graduate School of Chemical and Biological Sciences, The Scripps Research Institute, Jupiter, Florida, USA; 7 Department of Otorhinolaryngology-Head and Neck Surgery, Perelman School of Medicine, University of Pennsylvania 14640 , Philadelphia, Pennsylvania, USA; 8 Corporal Michael J. Crescenz VA Medical Center 20095, Philadelphia, Pennsylvania, USA; 9 Department of Infectious Diseases and Pathobiology, Vetsuisse Faculty, University of Bern 54179, Bern, Switzerland; 10 Institute for Virology and Immunology, Mittelhäusern and Bern, Bern, Switzerland; 11 Multidisciplinary Center for Infectious Diseases, University of Bern 27210, Bern, Switzerland; Duke University School of Medicine, Durham, North Carolina, USA

**Keywords:** alphacoronavirus, innate immunity, interferon, protein kinase R, ribonuclease L, HCoV-229E, HCoV-NL63

## Abstract

Compared with lethal betacoronaviruses, there is limited knowledge of how human alphacoronaviruses HCoV-NL63 (NL63) and HCoV-229E (229E) interact with host innate immune responses. We compared NL63 and 229E infections in human lung-derived cell lines, A549^ACE2^ and MRC-5, and primary nasal epithelial air-liquid interface (ALI) cultures. We measured the infection rates and viral replication kinetics. Additionally, we assessed the activation of three dsRNA-induced pathways, interferon (IFN) production and signaling, oligoadenylate synthetase-ribonuclease L (OAS/RNase L), and protein kinase R (PKR), following infection with each virus. Although both 229E and NL63 replicated efficiently in nasal ALI cultures, NL63 replicated minimally in A549^ACE2^ or MRC-5. In lung-derived cell lines, significant IFN mRNA induction as well as PKR activation was observed during NL63 but not during 229E infection. In contrast, in nasal ALI cultures, significant induction of both the IFN and PKR pathways was observed during 229E and NL63 infection. Notably, there was no evidence of RNase L activation during infection with either virus in cell lines or nasal ALI cultures. Infection with a recombinant 229E expressing an inactivated nsp15 endoribonuclease U (EndoU) led to increased dsRNA levels, stronger induction of all three antiviral pathways, and attenuation of replication relative to wild-type 229E. This indicates that 229E nsp15 EndoU regulates host dsRNA responses, as shown previously for porcine epidemic diarrhea virus (PEDV) and pathogenic betacoronaviruses. These findings demonstrate that NL63 and 229E differentially modulate host dsRNA-induced innate immune pathways and highlight the critical role of nsp15 EndoU in suppressing antiviral responses to facilitate efficient viral replication.

**IMPORTANCE:**

Seasonal human coronaviruses (HCoVs) are the causative agents of more than 15% of common cold cases each year. However, compared with more virulent HCoVs such as SARS-CoV-2, there has been limited research on these viruses. We compared the replication of HCoV-NL63 (NL63) and HCoV-229E (229E). Additionally, we examined their interactions with interferon signaling and related innate immune pathways in lung-derived cell lines and primary nasal epithelial cultures. 229E replicates efficiently in each of these culture systems, with significant dsRNA-induced pathway induction only in nasal cells. In contrast, NL63 replicates efficiently only in nasal cell cultures but induces innate immune pathways in all three culture systems. Moreover, the conserved CoV innate immune antagonist endoribonuclease U aids in evading these responses in 229E infection. This study expands our understanding of common-cold HCoV-host interactions and provides insight into differences between seasonal and lethal HCoVs.

## INTRODUCTION

Coronaviruses (CoVs), a family within the *Nidovirus* order, are enveloped, positive-sense, single-stranded RNA viruses (1, 2). To date, seven HCoVs have been identified, all of which are believed to be zoonotic, with origins in bats and mice (1, 3, 4). HCoVs are classified into either the betacoronavirus or alphacoronavirus genus. The betacoronaviruses include HCoV-OC43 (OC43), HCoV-HKU1 (HKU1), severe acute respiratory syndrome coronavirus (SARS-CoV), Middle East respiratory syndrome coronavirus (MERS-CoV), and SARS-CoV-2. The alphacoronaviruses include HCoV-NL63 (NL63) and HCoV-229E (229E) (5). 229E and NL63 are further classified into subgenera *Duvinacovirus* and *Setracovirus*, respectively (5). Although betacoronavirus-host interactions are well-studied, we will focus on the two human alphacoronaviruses, which have been relatively understudied. 229E was first isolated in 1967 from a patient with mild upper respiratory symptoms, whereas NL63 was isolated in 2004 from a 7-month-old infant suffering from bronchiolitis and conjunctivitis (6, 7). NL63 and 229E, together with betacoronaviruses OC43 and HKU1, circulate globally and are estimated to cause 15%–30% of mild-to-moderate upper respiratory tract illnesses in humans (5, 8). NL63 is also a leading cause of pediatric croup (laryngotracheobronchitis) (8–11), and both NL63 and 229E can trigger severe lower respiratory infections in vulnerable populations, including children, the elderly, and immunocompromised patients (12, 13).

**Fig 1:**
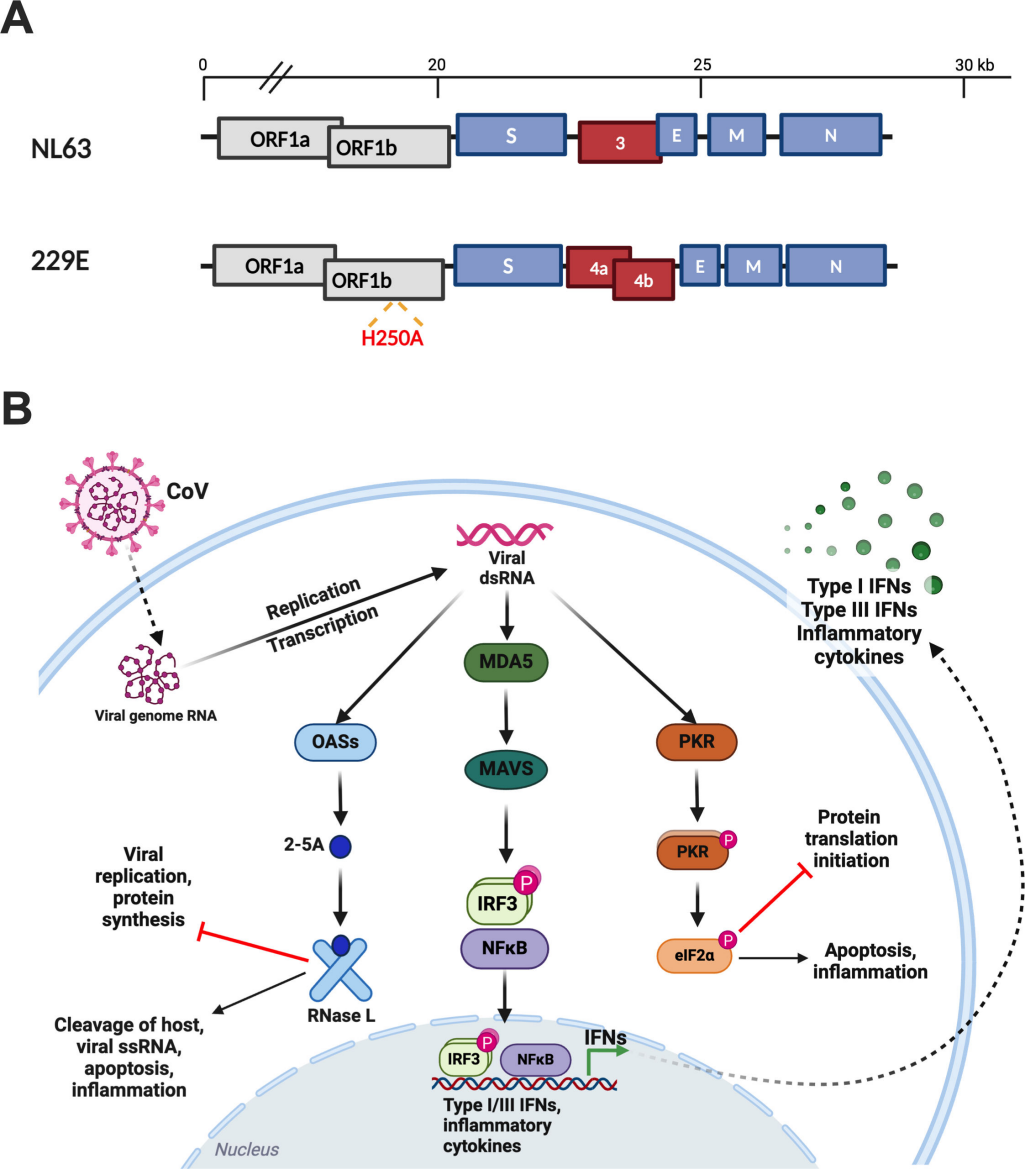
Human alphacoronavirus genomes and CoV dsRNA-induced innate immune responses. (**A**) The NL63 and 229E genome RNA with open reading frames (ORFs) are shown. The 5'-terminal two-thirds of the genome encodes two open reading frames (ORFs), ORF1a and ORF1b (gray). The 3' terminus encodes structural proteins (blue): spike (S), envelope (E), membrane (M), and nucleocapsid (N). Accessory genes are depicted in red. Red text indicates corresponding active site His to Ala substitution in the non-structural protein 15 within ORF1b of 229E EndoU-deficient mutant (r229E-nsp15^mut^). (**B**) During CoV infection, CoV dsRNA may be detected by cytosolic PRRs, leading to the production of IFN and subsequent ISG production, activation of the OAS/RNase L system, and activation of the protein kinase R (PKR) pathway. (Adapted from reference 16.) Graphics were created with BioRender.com.

All CoVs encode a single-stranded RNA genome with conserved organization. The 5′- two-thirds of the genome contains two overlapping open reading frames (ORFs), ORF1a and ORF1b, that encode 16 nonstructural proteins (nsp1–16). The remaining one-third of the genome contains ORFs encoding the viral structural proteins spike, envelope, membrane, and nucleocapsid, as well as accessory proteins that are distinct among HCoV genera and subgenera (1, 2, 5). CoV nonstructural proteins serve various roles in replication and transcription of CoV genomes and encode innate immune evasion functions. The 3’ accessory proteins have been shown to be dispensable for viral replication in most cell lines, although some play important roles in innate immune antagonism, viral pathogenesis, and virulence *in vivo* as well as in cell lines with intact innate immune responses (1, 2, 14). NL63 and 229E (laboratory-adapted versions) encode only one and two accessory proteins, respectively (Fig. 1A). This is in contrast to the more virulent MERS-CoV, SARS-CoV, or SARS-CoV-2, which each encode at least four accessory proteins (1, 3–5, 15).

CoV RNA replication takes place in replication transcription complexes (RTCs) within double-membrane vesicles (DMVs) formed from rearranged endoplasmic reticulum. As a byproduct of genome replication and subgenomic mRNA transcription, CoVs produce dsRNA, which serves as a pathogen-associated molecular pattern (PAMP) that is sensed by host cell pattern recognition receptors (PRRs), leading to activation of antiviral innate immune pathways. Thus, replication in DMVs is believed to protect the viral RNA from recognition by host sensors. Despite this shielding of CoV RNA, innate immune responses are initiated during coronavirus infection (17). Sensing of dsRNA by host melanoma differentiation-associated protein 5 (MDA5) (18) (a retinoic acid-inducible gene [RIG] I-like receptor [RLR]) (19) leads to the expression of type I and III IFNs and the downstream induction of IFN-stimulated genes (ISGs), cytokines, and chemokines (20–23). dsRNA is also recognized by protein kinase R (PKR), leading to its autophosphorylation and the subsequent phosphorylation of the eukaryotic translation initiation factor eIF2α, which shuts down protein synthesis (19, 24, 25). Oligoadenylate synthetases (OASs) serve as a third dsRNA sensor, producing 2’−5’-oligoadenylates, which activate the antiviral endoribonuclease RNase L, responsible for degrading both host and viral ssRNAs (26). These dsRNA-induced pathways are all independently activated; however, since PKR and OASs are themselves ISGs, both the PKR and OAS/RNase L pathways can be further induced by concurrent IFN signaling (19, 24, 25) (Fig. 1B).

In addition to the protection of RNA replication in DMVs, CoVs actively suppress innate immune responses by expressing both conserved nonstructural proteins (nsps) with host antagonist functions as well as genus/subgenus-specific accessory proteins (1, 17). Notable among these is the conserved nsp15 endoribonuclease (EndoU), which has previously been shown to act as an innate immune antagonist during infection with all four coronavirus genera (alpha, beta, gamma, and deltacoronaviruses) (27–36). CoV EndoU has been most thoroughly characterized in the murine coronavirus (MHV) model (30, 32, 37). EndoU is reported to cleave viral ssRNA, limiting the production of dsRNA byproducts and innate immune activation. Indeed, MHV recombinant viruses expressing enzymatically inactivated nsp15 produce more dsRNA, induce the IFN, PKR, and RNase L pathways more robustly, and are severely attenuated in primary macrophages as well as *in vivo* in the liver and spleen of mice relative to parental wild-type (WT) virus (30, 32). Similarly, we have recently reported that MERS-CoV and SARS-CoV-2 mutants expressing inactive nsp15 EndoU stimulate dsRNA-induced pathway activation and are attenuated for replication relative to WT in lung-derived cell lines as well as primary nasal epithelial cells (29, 38). Furthermore, recombinant 229E expressing an inactive nsp15 EndoU also exhibited a growth defect and elevated IFN-β production compared with WT 229E in human blood-derived macrophages (32), but the effect of nsp15 EndoU on 229E infection has not been assessed in respiratory epithelial cells.

Here, we compare 229E and NL63 in terms of their replication, induction of type I and type III IFN mRNA and ISGs, and activation of the PKR and OAS/RNase L pathways in human respiratory cell lines and primary nasal epithelial cell cultures grown at the air-liquid interface (ALI). These nasal ALI cultures model the initial site of viral replication in the human airway and the primary barrier to infection by respiratory viruses where innate immune responses are critical. We further characterize a 229E nsp15 mutant virus in terms of replication, dsRNA accumulation, and innate immune activation in the respiratory epithelium. These experiments contribute to our currently limited understanding of alphacoronavirus-host interactions.

## RESULTS

### NL63 and 229E differentially infect lung-derived cell lines

**Fig 2:**
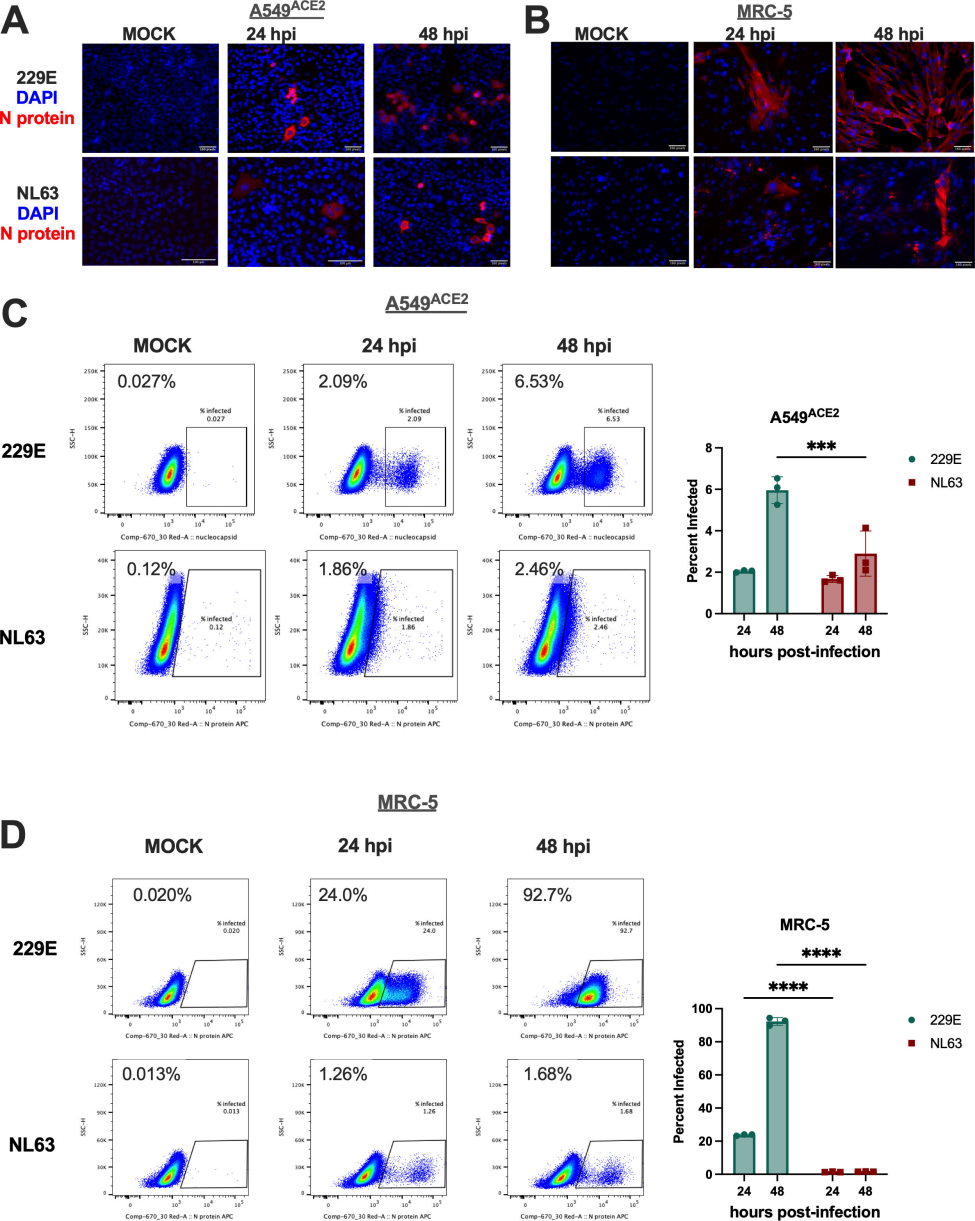
NL63 and 229E differentially infect lung-derived cell lines and primary nasal cells. (**A–D**) A549^ACE2^ or MRC-5 cells were mock-infected or infected with NL63 or 229E in triplicate at an MOI of 1. (A and B) At 24 or 48 hpi, indicated mock-infected and infected cells were fixed with 4% PFA and permeabilized; the expression of NL63 or 229E N protein (red) was examined by IF. Channels are merged with DAPI nuclear staining (blue). The images shown are representative of two independent experiments. (C and D) At the indicated time points, mock-infected or infected cells were harvested, stained with LIVE/DEAD fixable aqua dead cell stain, fixed and permeabilized, and stained intracellularly for the expression of NL63 or 229E N protein (APC). Representative flow cytometry plots with gating for *N*+ cells are shown to the left. Percent infected values were averaged from three independent technical replicates and displayed as mean ± SD to the right. These data are from one representative of two independent experiments. Statistical significance comparing 229E and NL63 at each time point was calculated by two-way ANOVA: *P  ≤  0.05; **P ≤ 0.01; ***P ≤ 0.001; ****P ≤ 0.0001. Data that were not statistically significant are not labeled.

We first evaluated the percentage of infected cells in the lung-derived epithelial cell line A549^ACE2^ (derived from a lung adenocarcinoma) (39) and the lung fibroblast cell line MRC-5 (derived from normal fetal lung tissue) during NL63 and 229E infection. A549^ACE2^ cells stably express angiotensin-converting enzyme 2 (ACE2); we used this cell type because ACE2 expression is required for the replication of NL63, and thus this allowed for comparison of 229E and NL63 in the same cell line (40, 41). To quantify the percentage of cells infected, cultures were infected with 229E or NL63 (multiplicity of infection [MOI] = 1 PFU/cell) at 33°C; this temperature was used for all experiments described below, as we and others have shown that common cold viruses such as NL63 and 229E replicate optimally at 33°C (nasal airway temperature) relative to 37°C (lower airway temperature) (42, 43). Cells were fixed at the indicated time points and stained with antibodies directed against 229E or NL63 nucleocapsid (N) protein for immunofluorescence (IF) or quantification via flow cytometry. Representative IF images are shown in Fig. 2A and B. In A549^ACE2^ cells, both 229E and NL63 infect relatively few cells at either time point (Fig. 2A). In MRC-5 cells, NL63 infects few cells while 229E infects nearly all cells by 48 h post-infection (hpi) (Fig. 2B). To obtain a more quantitative assessment of infection, the cells were fixed and stained with anti-NL63 or anti-229E N protein APC-conjugated antibody and analyzed by flow cytometry (see Fig. S1 for gating strategy). In A549^ACE2^ cells, 229E infection resulted in a slightly larger percentage of N-positive cells than NL63 at 48 hpi (6% vs 3%, respectively) (Fig. 2C). In MRC-5 cells, 229E infection led to a substantial increase in infected cells over time (~90% of cells infected by 48 hpi), whereas the percentage of infected cells following NL63 infection remained very low, consistent with IF data (Fig. 2D).

### 229E replicates more robustly than NL63 in respiratory cell culture

**Fig 3:**
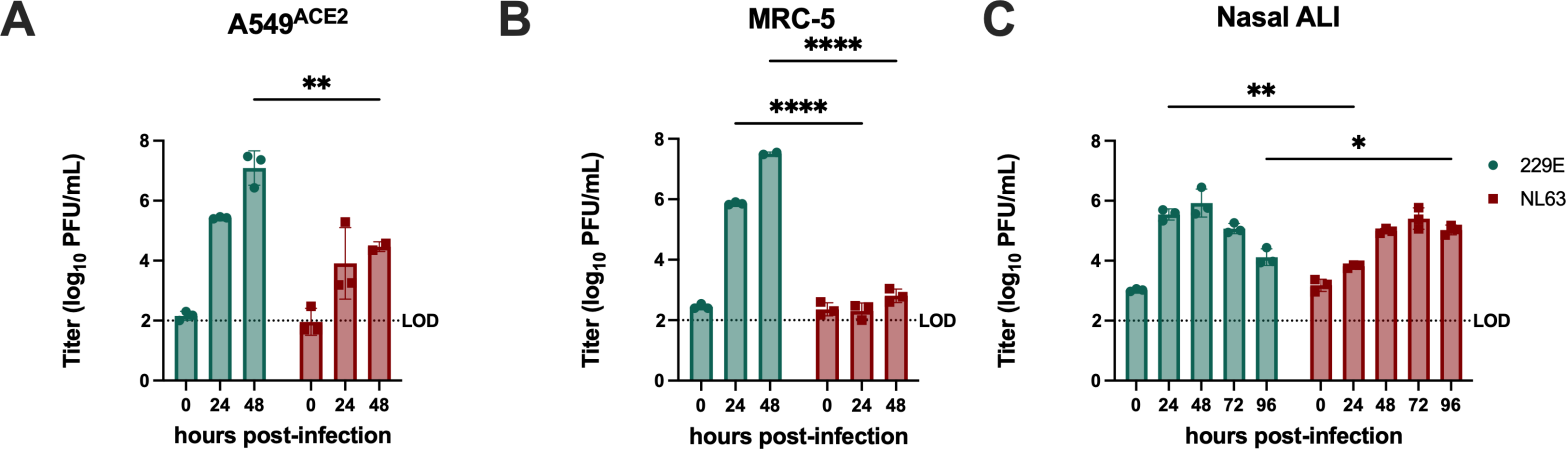
Replication kinetics distinguish NL63 and 229E. (**A–C**) Indicated cell type was infected with NL63 or 229E in triplicate at an MOI of 1, supernatant or apical surface liquid (ASL) was collected at the indicated time points, and infectious virus was quantified via plaque assay. (A and B) Titers from infected A549^ACE2^ or MRC-5 cells were averaged for each time point and depicted as mean ± standard deviation (SD) for each virus. (**C**) Titers from infected nasal ALI cultures. Each time point represents the averaged titer from three transwells, displayed as mean ± SD. Limit of detection (LOD) at 100 PFU/mL is indicated with a dotted line. Titer data are from one representative of three independent experiments. Statistical significance comparing 229E and NL63 at each time point was calculated by two-way ANOVA: *P  ≤  0.05; **P ≤ 0.01; ***P ≤ 0.001; ****P ≤ 0.0001. Data that were not statistically significant are not labeled.

We next compared the kinetics of replication of 229E and NL63 in A549^ACE2^ and MRC-5 cells. Cells were infected (MOI = 1 PFU/cell), and the infectious virus was collected from supernatants at 24 and 48 h post-infection (hpi) for quantification via plaque assay; peak titers were reached at 48 hpi, and a significant cytopathic effect was observed after 48 hpi. Despite the low percentage of infected cells seen for both viruses in A549^ACE2^ cells, 229E replicated efficiently to peak titer of 7.5 log_10_ PFU/mL at 48 hpi, whereas NL63 titers only slightly surpassed the plaque assay limit of detection (LOD) (Fig. 3A). In MRC-5 cells, 229E replicated more efficiently than NL63 (reaching titers of 7 log_10_ PFU/mL at 48 hpi), but NL63 replication was more efficient compared with A549^ACE2^ cells (reaching 4.5 log_10_ PFU/mL by 48 hpi) (Fig. 3B).

Since 229E and NL63 use different host cell receptors for viral entry (aminopeptidase N or APN for 229E and ACE2 for NL63), we hypothesized that the observed differences in replication in respiratory cell lines may be due to endogenous receptor expression levels (or, in the case of A549^ACE2^ cells, overexpressed receptor). We performed western blots on protein harvested from mock-infected A549^ACE2^ and MRC-5 cells (Fig. S2). Our results indicate that receptor expression level is not the primary determinant of HCoV replication in these cell lines. APN expression was significantly higher in MRC-5 than in A549^ACE2^ cells, which may explain the increased percentage of infected cells in MRC-5 (Fig. 2D) but not the high 229E titers in both cell lines. ACE2 expression is high in A549^ACE2^ cells and nearly undetectable in MRC-5 cells, and thus, receptor expression levels do not correlate with NL63 replication or proportion of infected cells.

We have previously optimized a nasal epithelial culture system to model viral replication and innate immune induction at the primary barrier site to infection. Primary nasal cells are differentiated at an air-liquid interface (ALI) to recapitulate the heterogeneous cellular population and mucociliary function of the nasal airway. (42, 44, 45). Nasal ALI cultures were infected at an MOI of 5 PFU/cell to maximize the percentage of cells initially infected, which we have previously reported to be low (45), and apical surface liquid (ASL) was collected at 24-h intervals for quantification of shed virus titers via plaque assay. Growth curves in nasal ALI cultures were extended to 96 hpi, based on our previous observations of peak viral titers in this system (42, 45). In contrast to replication data in lung-derived cell lines, both 229E and NL63 replicated efficiently in nasal ALI cultures (peak titers ~ 6 log_10_ PFU/mL, although with slightly different kinetics) (Fig. 3C). Receptor expression level in nasal ALI cultures similarly does not predict replication, as ACE2 levels are lower than in A549^ACE2^ (despite efficient NL63 replication). Taken together, our data highlight the limited replication of NL63 in lung-derived cell lines relative to 229E and suggest that primary epithelial cell culture systems may be required to adequately compare these viruses.

### NL63 and 229E differentially induce the interferon (IFN) and protein kinase R (PKR) pathways

**Fig 4:**
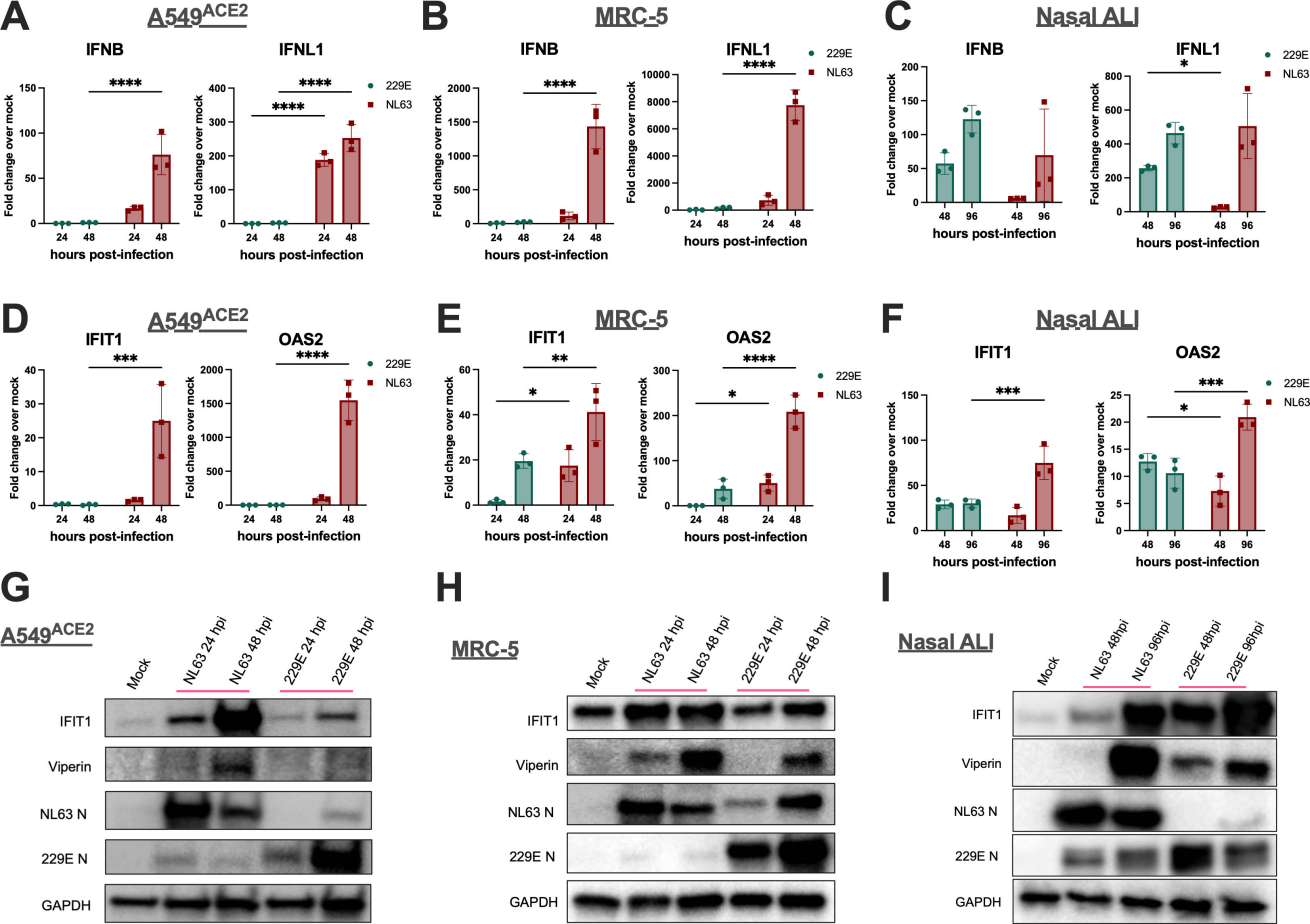
NL63 and 229E differentially induce IFN and ISG expression. (**A–F**) Indicated cell types were mock-infected or infected in triplicate at MOI = 5 with NL63 or 229E. Total RNA was harvested at the indicated time points, and *IFNB*, *IFNL1*, *IFIT1*, or *OAS2* mRNA expression was quantified by qRT-PCR. C_T_ values were normalized to 18S rRNA and expressed as fold-change over mock, displayed as 2^-Δ(ΔCt)^. Each point represents averaged mRNA expression from three independent wells or transwells, displayed as mean ± SD. (**G–I**) Indicated cell type was mock-infected or infected in triplicate at MOI = 5 with NL63 or 229E. Cell lysates were harvested at the indicated times, and proteins were separated by SDS/PAGE and immunoblotted with antibodies against IFIT1, Viperin, NL63 N, 229E N, and GAPDH. Data are from one representative of two independent experiments. Statistical significance comparing 229E and NL63 at each time point was calculated by two-way ANOVA: *P  ≤  0.05; **P ≤ 0.01; ***P ≤ 0.001; ****P ≤ 0.0001. Data that were not statistically significant are not labeled.

To investigate the degree of IFN activation during infection with 229E or NL63, cells were infected (MOI = 5), and intracellular RNA was extracted following cell lysis at the indicated time points. RT-qPCR was used to quantify the mRNA expression of type I/III IFN genes (*IFNB* and *IFNL1*) and representative ISGs (*IFIT1* and *OAS2*). In A549^ACE2^ cells, no significant induction of IFN or ISGs was observed during 229E infection at either time point relative to mock-infected cells (Fig. 4A and D). In contrast, infection of A459^ACE2^ cells with NL63 robustly induced *IFNB* and *IFNL1* as well as *IFIT1* and *OAS2* by 48 hpi (Fig. 4A and D). As in A549^ACE2^, we observed more IFN and ISG mRNA induction during NL63 infection of MRC-5 cells, compared with 229E infection (Fig. 4B and E). We further examined ISG expression at the protein level via western blot with antibodies against IFIT1 and Viperin. ISG protein expression was greatest during NL63 infection of both cell types, with minimal induction during 229E infection, corroborating our results at the mRNA level (Fig. 4G and H). We performed similar experiments in nasal ALI cultures, in which both viruses replicate efficiently (42, 45). IFN and ISG mRNA expressions, as well as ISG protein expression, were induced significantly following either 229E or NL63 infection, with increased activation at the later time point (Fig. 4C, F, and I).

**Fig 5:**
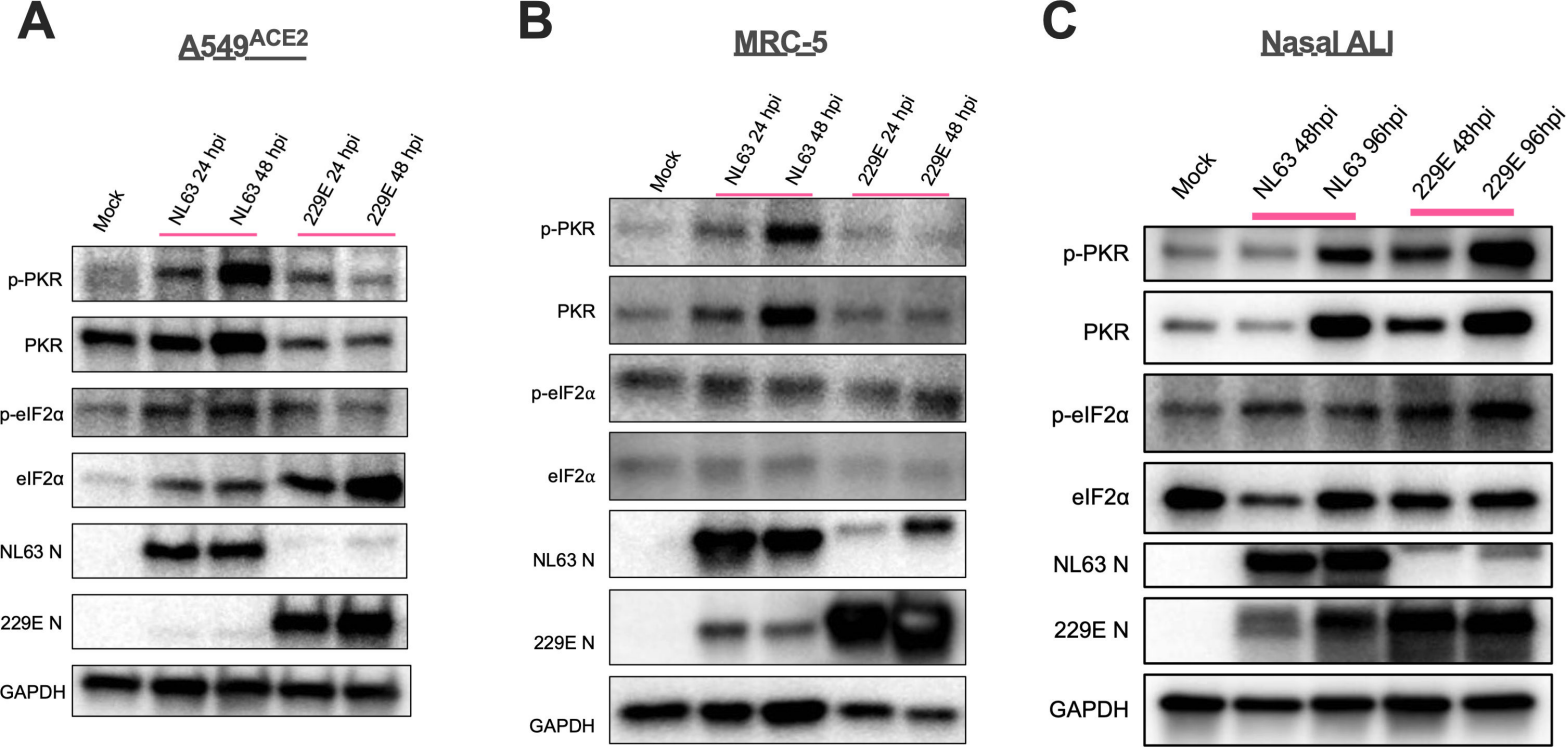
NL63 and 229E induce activation of the PKR pathway. Indicated cell type was mock-infected or infected in triplicate at MOI = 5 with NL63 or 229E. (**A–C**) Cell lysates were harvested at the indicated times, and the proteins were separated by SDS/PAGE and immunoblotted with antibodies against phosphorylated PKR (p-PKR), PKR, phosphorylated eIF2α (p-eIF2α), eIF2α, NL63 N, 229E N, and GAPDH. Data are from one representative of two independent experiments.

Next, we analyzed 229E- and NL63-infected protein lysates via western blot for activation of the PKR pathway using antibodies against phosphorylated PKR (p-PKR) and its downstream target eIF2α (p-eIF2α). In line with the IFN pathway data, we observed PKR phosphorylation in NL63-infected A549^ACE2^ and MRC-5 cells, whereas only mild p-PKR above mock-infected levels was observed during 229E infection (Fig. 5A and B). Total PKR levels were also increased during NL63 infection, which was expected given that PKR is an ISG. Phosphorylation of downstream target eIF2α occurred only during NL63 infection in A549^ACE^ cells, whereas p-eIF2α levels were not increased above mock during NL63 infection of MRC-5 cells or during 229E infection of either cell line (Fig. 5A and B), suggesting incomplete activation of the PKR pathway or phosphorylation of eIF2α that is below the level of detection via western blot. A similar analysis of protein samples from nasal ALI cultures revealed p-PKR activation by both 229E and NL63, contrasting once more with data in cell lines. Interestingly, we observed stronger and earlier induction of p-PKR in 229E-infected nasal cells relative to NL63 (Fig. 5C). p-eIF2α was also induced in nasal ALI cultures following 229E but not NL63 infection, consistent with our previous observation that p-eIF2α is difficult to detect over background levels in nasal cultures (46). We also note that there is some cross-reactivity between antibodies against 229E and NL63 N protein, as we have observed previously. This is not surprising as the genomes of these two strains share some sequence homology (47).

We next investigated whether infection with 229E or NL63 induced the activation of the OAS/RNase L pathway, using degradation of ribosomal RNA (rRNA) as a readout for RNase L activity. Infection with Sindbis virus (SINV), an alphavirus that robustly activates RNase L, served as a positive control with robust degradation of 18S and 28S rRNA (16, 48, 49). rRNA remained intact in 229E- and NL63-infected A549^ACE2^ or MRC-5 cells, suggesting that RNase L was not activated by either virus (Fig. S3A and B). There was also no detectable rRNA degradation observed following infection of nasal ALI cultures with either virus, although it is important to note that we have never detected RNase L activation in the nasal ALI culture system (Fig. S3C). Overall, we did not observe activation of the RNase L pathway in any cell type infected with NL63 and 229E.

### 229E nsp15 EndoU antagonizes dsRNA-induced antiviral pathways

Our data highlight relatively minimal induction of the IFN, PKR, and OAS/RNase L pathways during 229E infection, except during infection of nasal ALI cultures. Prior studies have identified the conserved CoV nsp15 endoribonuclease as a potent inhibitor of dsRNA-induced immune pathways during infection by multiple viruses, including betacoronaviruses MHV, MERS-CoV, and SARS-CoV-2, and alphacoronavirus PEDV. We sought to explore the role of nsp15 EndoU during 229E infection using a recombinant 229E expressing a catalytically inactivated nsp15 EndoU (His to Ala mutation at amino acid residue 250) (Fig. 1A). This recombinant virus was previously characterized by Kindler et al. in human blood-derived macrophages (32) but has not been characterized in airway culture systems. We compared the extent of replication, percentage of infected cells, as well as innate immune induction following infection with the isogenic recombinant wild-type (WT) 229E (r229E) vs. r229E-NSP15^mut^ (32, 50). These recombinant viruses contain eight amino acid substitutions when compared with the 229E virus used in the above experiments (see Materials and Methods). We chose to use MRC-5 cells and nasal ALI cultures to characterize these viruses, forgoing A549^ACE2^ cells, as NL63 (which requires the ACE2 receptor) was not included in these experiments. Additionally, in contrast to our laboratory 229E strain, these recombinant 229E strains failed to replicate in A549^ACE2^.

**Fig 6:**
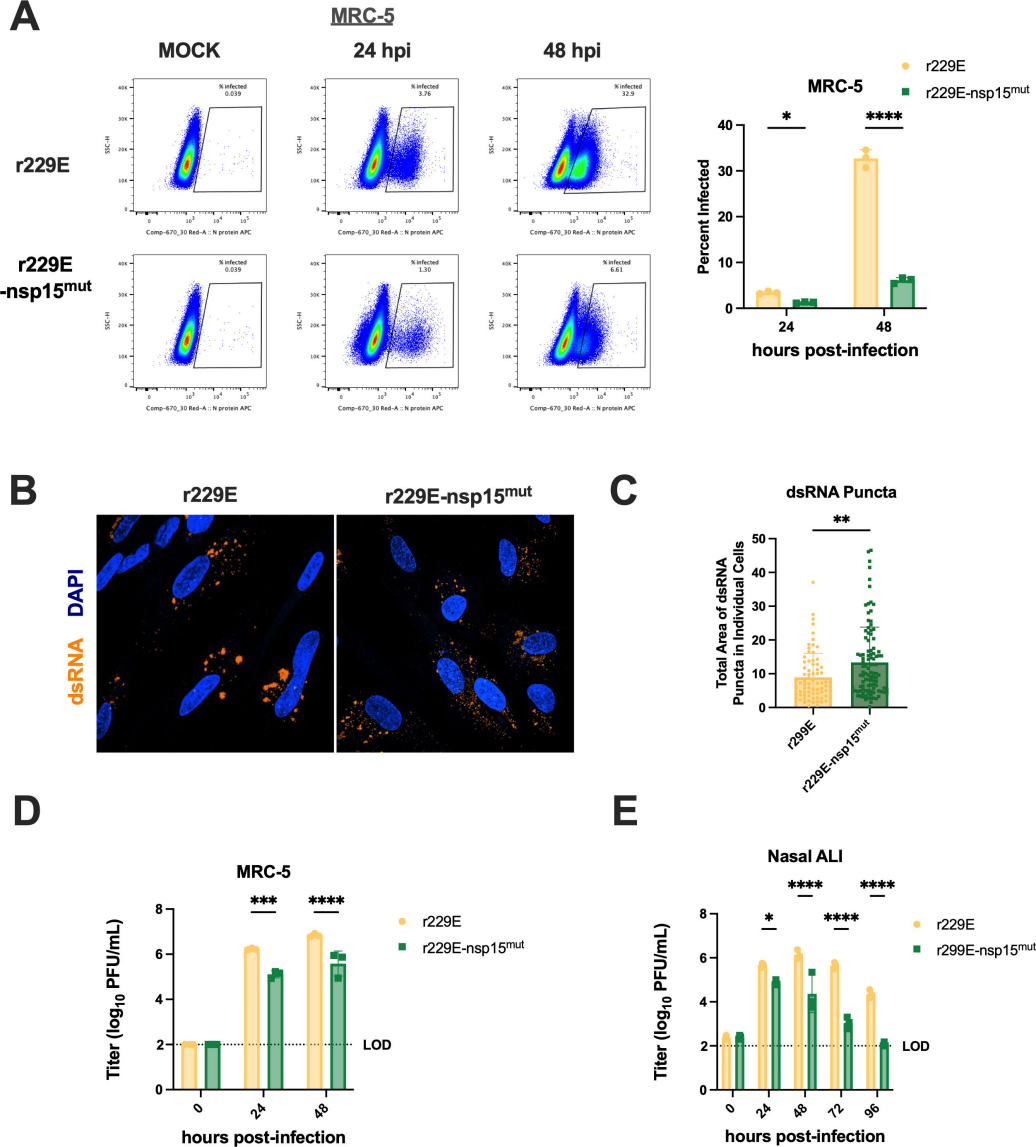
Nsp15 EndoU-deficient 229E infects fewer cells, produces increased dsRNA, and is attenuated relative to wild-type 229E. (**A**) At indicated time points, mock-infected or infected (MOI = 1) MRC-5 cells were harvested, stained with LIVE/DEAD fixable aqua dead cell stain, fixed and permeabilized, and stained intracellularly for the expression of 229E N protein (APC). Representative flow cytometry plots with gating for *N*+ cells are shown to the left. Percent infected values were averaged from three independent technical replicates and displayed as mean ± SD to the right. These data are from one representative of two independent experiments. (**B**) Representative images of infected MRC-5 cells (MOI = 5) fixed at 48 hpi, stained for dsRNA using K1 antibody, and nuclei visualized with DAPI. (**C**) Quantification of the total area of dsRNA puncta in MRC-5 cells at 48 hpi. Each point represents the total area of dsRNA signal in a single cell. Data shown are from three fields of view quantified in two independent experiments. (**D**) Titers from MRC-5 cells infected with the indicated virus (MOI = 1) were averaged for each time point and depicted as mean ± standard deviation (SD) for each virus. (**E**) Titers from nasal ALI cultures infected with the indicated virus (MOI = 5). Each time point represents the averaged titer from three transwells, displayed as mean ± SD. Limit of detection (LOD) at 100 PFU/mL is indicated with a dotted line. Titer data are from one representative of two independent experiments. Statistical significance compared with WT 229E was calculated by two-way ANOVA: *P  ≤  0.05; **P ≤ 0.01; ***P ≤ 0.001; ****P ≤ 0.0001. Data that were not statistically significant are not labeled.

We first quantified the percentage of infected cells following the infection of MRC-5 cells with r229E and r229E-nsp15^mut^ via flow cytometry and found a significant decrease in the proportion of N-positive cells during nsp15^mut^ relative to WT infection. This difference was largest at the later time point (33% vs 6% infected for WT vs nsp15^mut^, respectively) (Fig. 6A). Given the proposed mechanism of nsp15 EndoU in limiting dsRNA accumulation during infection, we quantified dsRNA production during infection of MRC-5 cells with either virus. Representative images are shown in Fig. 6B, in which there appeared to be a larger number of smaller dsRNA puncta dispersed throughout infected cells following nsp15 mutant infection. Quantification of dsRNA levels revealed a significantly increased total area of dsRNA puncta per cell following r229E-nsp15^mut^ relative to r229E infection (Fig. 6C). We additionally separated individual dsRNA puncta according to area (small, medium, and large) and found an increased number of small puncta (<1.0 µm^2^) during r229E-nsp15^mut^ infection (Fig. S4A), whereas more large puncta (>5.0 µm^2^) were observed during r229E infection (Fig. S4B). This suggests that dsRNA is more dispersed throughout the cell during infection with the mutant virus.

We next evaluated the replication kinetics of these viruses in MRC-5 as well as nasal ALI cultures. Consistent with the lower percentage of infected cells, r229E-nsp15^mut^ is attenuated relative to r229E in MRC-5 cells by ~1 log_10_ PFU/mL (Fig. 6D). This growth defect was even more pronounced in nasal ALI cultures, in which nsp15^mut^ was 1-3 log_10_ PFU/mL attenuated (depending on time point), with mutant virus titers diminishing to nearly the limit of detection by 96 hpi (Fig. 6E). This suggests that earlier and more efficient viral clearance following r229E-nsp15^mut^ relative to r229E infection in nasal cultures.

**Fig 7:**
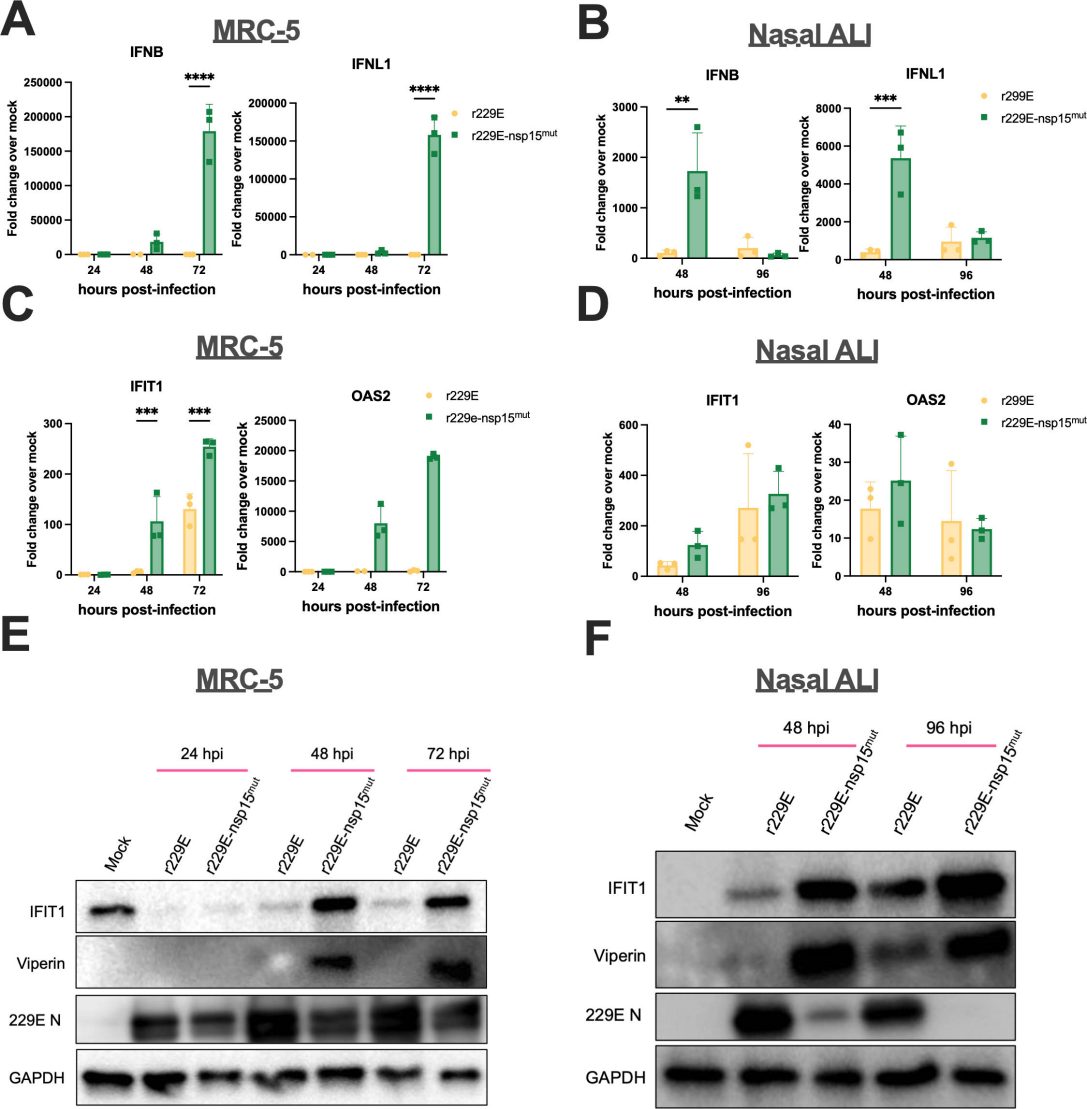
EndoU-deficient 229E induces IFN signaling more robustly than WT. (**A-D**) Indicated cells were mock-infected or infected in triplicate at MOI = 5 with r229E or r229E-nsp15^mut^. Total RNA was harvested at indicated time points, and *IFNB*, *IFNL1*, *IFIT1*, or *OAS2* mRNA expression was quantified by qRT-PCR. C_T_ values were normalized to 18S rRNA and expressed as fold-change over mock, displayed as 2^-Δ(ΔCt)^. Each point represents averaged mRNA expression from three independent wells or transwells, displayed as mean ± SD. Data are from one representative of two independent experiments. Statistical significance compared to WT 229E was calculated by two-way ANOVA: *P  ≤  0.05; **P ≤ 0.01; ***P ≤ 0.001; ****P ≤ 0.0001. Data that were not statistically significant are not labeled. (**E and F**) Indicated cells were mock-infected or infected in triplicate at MOI = 5 with r229E or r229E-nsp15^mut^. Cell lysates were harvested at the indicated times, and the proteins were separated by SDS/PAGE and immunoblotted with antibodies against phosphorylated IFIT1, Viperin, NL63 or 229E N, and GAPDH. Data are from one representative of two independent experiments.

**Fig 8:**
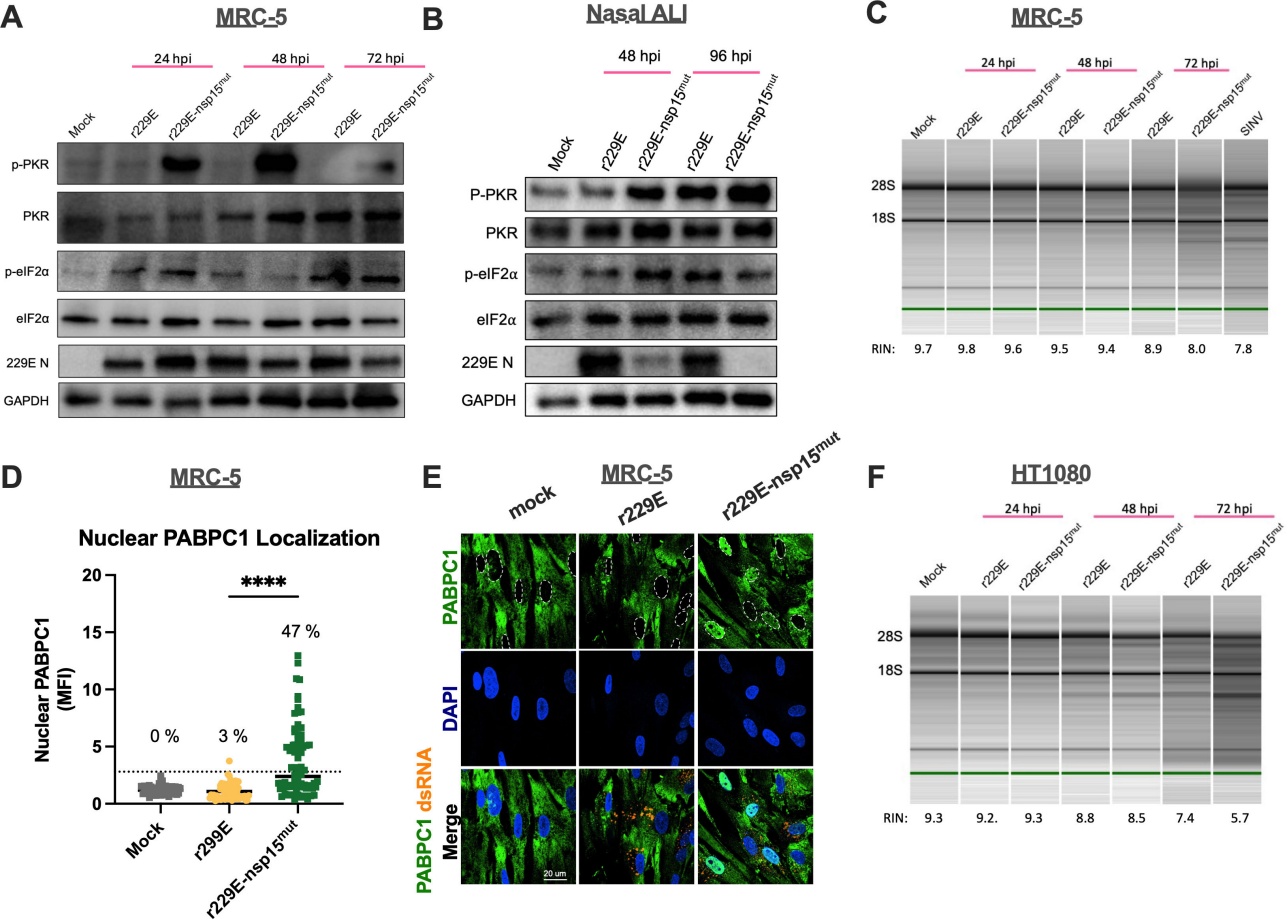
229E-nsp15^mut^ robustly induces the PKR and OAS/RNase L pathways. MRC-5, nasal ALI cultures, or HT1080 cells (as indicated) were mock-infected or infected in triplicate at MOI = 5 with r229E or r229E-nsp15^mut^. (**A and B**) Cell lysates were harvested at the indicated times, and proteins were separated by SDS/PAGE and immunoblotted with antibodies against phosphorylated PKR (p-PKR), PKR, phosphorylated eIF2a (p-eIF2α), eIF2α, 229E N, and GAPDH. Data are from one representative of two independent experiments are shown. (**C**) Total cellular RNA was harvested from infected MRC-5 cells at the indicated times post-infection. rRNA degradation was assessed on an Agilent Bioanalyzer. 28S and 18S rRNA positions are indicated. Data are from one representative of two independent experiments. (**D**) IF assay for dsRNA (K1 antibody; yellow) and PABPC1 (green) in MRC-5 cells. Nuclei were stained with DAPI (blue). Quantification of PABPC1 nuclear localization is shown, with the percentage indicating proportion of cells with nuclear PABPC1 over the highest mock value in infected cells. Data shown are from three fields of view quantified in two independent experiments. (**E**) Representative IF images for PABPC1 (green), dsRNA (yellow), or DAPI (blue). The white outlines in the PABPC1 panel indicate the nuclei of each cell. (**F**) Total cellular RNA was harvested from infected HT1080 cells at the indicated times post-infection, and rRNA degradation was assessed on an Agilent Bioanalyzer. RNA integrity numbers (RIN) are shown under each lane in panels C and F, with lower RIN indicating increased RNA degradation. Data are from one representative of two independent experiments.

We hypothesized that the increased dsRNA production during infection with the 229E nsp15 mutant would result in stronger induction of dsRNA-induced pathways, which would explain its replication defect as well as limited spread of infection (and lower percentage of infected cells). In both MRC-5 cells and nasal ALI cultures, we found that type I and III IFN mRNAs were induced more robustly during r229E-nsp15^mut^ relative to r229E infection (Fig. 7A and B). Representative ISG mRNA levels (IFIT1, OAS2) were also increased during infection with the nsp15 mutant in MRC-5 cells, but were induced to a similar extent in nasal ALI cultures (Fig. 7C and D). This increased IFN signature during nsp15^mut^ infection was also observed at the protein level in both cell types using western blots for IFIT1 and Viperin protein expression (Fig. 7E and F). When we compared these viruses in terms of induction of the PKR pathway, we observed increased PKR phosphorylation during r229E-nsp15^mut^ infection relative to r229E in both MRC-5 and nasal ALI cultures (Fig. 8A and B). We did not detect a significant increase in p-eIF2α signal during nsp15 mutant infection in either cell type. Corroborating our replication data, there was a clear decrease in 229E N levels during infection with r229E-nsp15^mut^ infection compared with r229E in nasal ALI culture, with 229E N being nearly undetectable at late times during infection with the mutant (Fig. 8B).

Finally, we evaluated rRNA degradation patterns as a readout for RNase L activation and found that r229E-nsp15^mut^ infection of MRC-5 cells resulted in RNase L activation at 72 hpi, whereas significant activation of this pathway was not detected during parental r229E infection (Fig. 8C). Since this was our first observation of RNase L activity during alphacoronavirus infection, we assessed the percentage of infected cells that activated RNase L by IF assays for dsRNA (infection marker) and poly[a]-binding protein-1 (PABPC1), which translocates from the cytosol to the nucleus upon activation of RNase L-mediated mRNA decay (51–53). We observed that 47% of cells infected with r229E-nsp15^mut^ displayed nuclear PABPC1 staining (Fig. 8D and E), whereas only 3% of cells infected with r229E displayed nuclear PABPC1. These data show that 229E nsp15 inhibits the activation of RNase L. We have previously used a fibrosarcoma cell line, HT1080, to evaluate RNase L activity, so we infected HT1080 cells with r229E and r229E-nsp15^mut^ and evaluated rRNA integrity. Confirming our results in MRC-5 cells, RNase L was activated following infection with the nsp15 mutant in HT1080 cells (Fig. 8F). RNase L was not activated during infection with either r229E or r229E-nsp15^mut^ in nasal ALI cultures, which was not surprising as we have never detected RNase L activation in these cultures (Fig. S3D). These data highlight nsp15 as a potent antagonist of dsRNA-induced immunity during 229E infection.

## DISCUSSION

Although interest in lethal coronaviruses has intensified in recent years, driven by the global impact of the SARS-CoV-2 pandemic and COVID-19 disease, the seasonal coronaviruses, NL63, 229E, OC43, and HKU1, have not received extensive research attention in part due to challenges associated with traditional cell culture systems. We have recently reported methods for the propagation and quantification of OC43, 229E, and NL63 by identifying the optimal infection temperature (33°C) at which immortalized cell lines should be used to generate high-titer virus stocks and for optimal virus titration for each seasonal HCoV (43). In addition, we recently compared the cellular tropism, replication kinetics, and virus-induced cytotoxicity of lethal and seasonal HCoVs in nasal ALI cultures at 33°C (44, 45). Tissue culture models for the study of pathogenic HCoVs such as MERS-CoV and SARS-CoV-2 have been extensively characterized, and these viruses’ ability to evade and suppress host antiviral pathways to optimize their replication has been reported by our group and others (16, 29, 54–57). In contrast, there are relatively few studies that compare airway models for the characterization of NL63 and 229E and their activation or evasion of dsRNA-induced antiviral responses. To gain a more comprehensive understanding of the entire HCoV family, we characterized these human alphacoronaviruses and their interactions with dsRNA-induced immune pathways using three respiratory culture systems, the lung epithelial A549^ACE2^ cell line, the lung fibroblast MRC-5 cell line, and primary nasal epithelial ALI cultures (43, 44).

In comparing the replication kinetics and percentage of cells infected by each virus (Fig. 2 and 3), we found that 229E robustly replicates in A549^ACE2^, MRC-5, and nasal ALI cultures, whereas NL63 only reaches high viral titers in nasal ALI cultures. This suggests that primary cell culture systems may be necessary in order to compare alphacoronaviruses. We speculate that this may be because NL63 requires features of the *in vivo* airway, such as a heterogeneous cellular population and mucociliary function, for optimal viral entry and replication. Our prior experiments with NL63 have identified ciliated epithelial cells as the primary cell type infected by NL63 in nasal ALI culture (45). Additionally, we observed that 229E infected a much smaller percentage of A549^ACE2^ cells relative to MRC-5 cells but achieved high viral titers in both cell types without induction of significant innate immune responses. We cannot explain the surprising observation of production of high titers of 229E despite the low percentage of infected A549 cells; we hypothesize that intracellular events apart from innate immunity may regulate the level of viral replication on a per-cell basis, allowing for high viral titers produced by a low percentage of infected cells.

It is important to note that these two viruses use different receptors for entry into host cells. NL63 utilizes angiotensin-converting enzyme-2 (ACE2), whereas 229E uses aminopeptidase N (APN) (40, 58–60). When we compared expression of these cellular receptors in each of the three airway culture systems (Fig. S2), we found that receptor expression is not the major determinant of viral replication. ACE2 expression was highest in A549^ACE2^ cells, but NL63 failed to replicate efficiently in this cell line. ACE2 expression was comparatively below the limit of detection by western blot in nasal ALI cultures, in which NL63 replicated robustly. These data suggest that high levels of the cellular receptor are not necessary for productive infection, as we have demonstrated previously for SARS-CoV-2 (16) and MHV infections (61). The inability of NL63 to replicate efficiently in A549^ACE2^ cells may be due to robust host responses (such as IFN/ISG induction) that limit viral replication and spread. Alternatively, there may be co-receptors or other cellular factors required for efficient infection in cell culture. For example, ACE2 plays a critical role in SARS-CoV-2 replication; however, ACE2 expression profiles along the airway are not always directly associated with infection patterns, and ACE2-independent alternative receptors have been reported to mediate SARS-CoV-2 entry (62). Potential alternative mechanisms for NL63 entry (which may be particularly important for tissue culture-adapted strains of NL63) have yet to be reported.

We report that NL63 significantly induced IFN signaling and PKR pathway activation in both A549^ACE2^ and MRC-5 cells despite low levels of replication, whereas 229E did not appreciably induce either pathway in these cell lines (Fig. 4 and 5). Conversely, in nasal ALI cultures, both 229E and NL63 induce the IFN/ISG and PKR pathway, with earlier activation during 229E infection (Fig. 4 and 5). We previously observed early induction of antiviral interferon (IFN) signaling during 229E and NL63 infection of nasal ALI cultures and showed that clearance of both viruses from these cultures was IFN-mediated (46). Consistent with our findings but limited to 229E, two additional studies confirm induction of the IFN pathway following 229E infection, in bronchial epithelial ALI cultures and MRC-5 cells (63, 64).

Although we observed PKR phosphorylation during NL63 infection of all three cell types and during 229E infection of nasal cell cultures, we did not consistently observe phosphorylation of its downstream mediator, eIF2a. This may be partially related to the fact that PKR is itself an ISG and is thus upregulated in the context of IFN signaling, whereas eIF2α is not induced by IFN. Additionally, PKR is not the sole kinase that phosphorylates eIF2α, but rather one of four kinases that compose the integrated stress response (ISR) (65, 66). PKR-like ER kinase (PERK), general control nondepressible 2 (GCN2), and heme-regulated eIF2α kinase (HRI) can also activate eIF2α, following the accumulation of unfolded proteins, amino acid starvation, and heme deficiency, respectively (65, 66). In addition, host pathways antagonizing phosphorylation of eIF2α may be activated during alphacoronavirus infection. For example, we previously reported that GADD34 activation during infection with betacoronaviruses OC43 and MERS-CoV results in dephosphorylation of eIF2α (67). Future experiments will evaluate translational shutoff (the downstream impact of PKR pathway activation), and the extent to which these additional kinases and regulators of eIF2α phosphorylation may or may not be activated during 229E and NL63 infection.

This limited activation of the IFN and PKR pathway during infection of cell lines with 229E led us to investigate innate immune antagonism during 229E infection. The conserved CoV nsp15 EndoU has been reported to serve as a potent inhibitor of dsRNA-induced pathways during infection of all four CoV genera: betacoronavirus MHV, SARS-CoV-2, MERS-CoV, alphacoronavirus PEDV infection (27–34), as well as gammacoronavirus infectious bronchitis virus (IBV) (35) and porcine deltacoronavirus (PDCoV) (36). A 229E recombinant mutant expressing a defective nsp15 protein had previously been shown to be attenuated in human macrophages (32) but has not been characterized in airway culture systems. Quantification of dsRNA production during infection with the 229E nsp15^mut^ revealed an increase in total dsRNA production in individual infected cells relative to WT (Fig. 6B), as well as an increase in the number of small dsRNA puncta. Our findings seem to corroborate a model whereby mutation of nsp15 EndoU results in increased dispersal of dsRNA throughout the cytoplasm, as has been suggested during MHV infection (68). This increase in dsRNA resulted in increased dsRNA-induced pathway activation as well as attenuated replication during r229E-nsp15^mut^ infection relative to WT (Fig. 6D, E, 7, and 8).

Interestingly, infection with this 229E nsp15 mutant resulted in activation of the OAS/RNase L pathway, as evidenced by total rRNA degradation in MRC-5 and HT1080 cells (Fig. 8C and F), as well as increased nuclear PABPC1 localization in MRC-5 cells (Fig. 8D and E). This is the first instance of activation of this pathway in the context of alphacoronavirus infection, as neither WT 229E nor NL63 activated OAS/RNase L in any cell culture system. Studies of betacoronaviruses have highlighted that SARS-CoV-2 induces RNase L in A549^ACE2^ and Calu3 cells (16, 28), whereas MERS-CoV infection only results in RNase L activation when two of its innate immune antagonists are inactivated (nsp15 EndoU and NS4b, an accessory protein with phosphodiesterase activity) (29). We have previously failed to observe RNase L activation in nasal ALI cultures during a variety of RNA virus infections or via treatment with synthetic dsRNA, poly(I:C) (28), suggesting that this cell type may be unable to activate the pathway. Indeed, we have observed previously that several primary mouse cell types are also unable to activate this pathway due to insufficient expression levels of OAS genes (the sensor responsible for OAS/RNase L activation) (69–71). We do not have an EndoU-deficient NL63 mutant available for study, but we hypothesize that infection with such a virus would result in significant attenuation of the virus, which would be most striking in nasal ALI culture, where NL63 replicates most efficiently. Studying the role of EndoU during NL63 infection would provide a better understanding of similarities and differences between human alpha- and beta-coronavirus nsp15 activity.

In addition to the conserved CoV nsp15 EndoU, accessory proteins likely contribute to the differential innate immune activation observed in response to common cold alphacoronaviruses compared with lethal betacoronaviruses. Indeed, 229E and NL63 have the smallest genome sizes of the seven HCoVs, each approximately 27.5 kb (5), and as such contain fewer accessory genes than the lethal betacoronaviruses that contain numerous accessory genes (at least four for SARS-CoV-2 and MERS-CoV) that serve as innate immune antagonists (29, 54). NL63 encodes only one presumed accessory protein, encoded in ORF3. The NL63 ORF3 protein was initially thought to function similarly to SARS-CoV ORF3a, which was shown to play a role in the regulation of NFκB-dependent cytokines and modulation of S protein-mediated endocytosis (72). Müller et al. reported that the NL63 ORF3 protein colocalizes with E and M within the endoplasmic reticulum/Golgi intermediate compartment (ERGIC) and that it is N-glycosylated at the N-terminus. Analysis of purified viral particles revealed that the ORF3 protein is incorporated into virions and, therefore, is an additional structural protein with a proposed function within the viral assembly and budding process (73), but its specific role during NL63 function has not been characterized. The prototype laboratory-adapted 229E strain has a split accessory gene, encoding the putative ORF4a and ORF4b proteins (74). ORF4a localizes to the ERGIC in infected cells and possesses ion channel activity, demonstrated in both Xenopus oocytes and yeast (75). Interestingly, an analysis of five 229E clinical isolates found that each encodes ORF4, which is a homolog of the NL63 ORF3 protein, instead of ORF4a and ORF4b proteins encoded by MERS-CoV (74), but its function has not been thoroughly characterized. Overall, there are limited data implicating NL63 and 229E accessory proteins in antagonizing innate immune responses. To understand the function of these accessory proteins during authentic infection, it will be necessary to generate and characterize recombinant viruses lacking each of these genes, as we and others have done previously with MERS-CoV and SARS-CoV-2 accessory genes (29, 54).

With the potential for new pathogenic HCoVs to emerge, there is a need to fully characterize and understand all HCoVs and their host interactions to guide surveillance and aid the creation of antiviral treatments and vaccines. By characterizing human alphacoronaviruses in multiple airway culture systems, we begin to understand the virus-host interactions that may help predict pathogenesis and transmissibility. Future studies will compare alphacoronaviruses in upper (nasal) vs. lower (bronchial) airway culture systems, as well as compare recombinant alphacoronaviruses lacking their accessory genes with similar betacoronavirus mutants.

## MATERIALS AND METHODS

### Viruses

Our laboratory strain NL63 and 229E genome sequences were compared with wild-type (WT) reference sequences, NL63 (ATCC NR-470; GenBank: AY567487 or NC_005831.2) and 229E (ATCC VR-740; GenBank NC_002645), respectively. Nucleotide homologies between our laboratory strain and reference strain were 99.93% for 229E and 99.96% for NL63. The recombinant wild-type 229E (r229E) and nsp15 EndoU-deficient mutant virus (r229E-nsp15^mut^) were generated using the vaccinia virus-based reverse genetic system as previously described (32, 50, 76). The r229E genome is 100% identical to GenBank NC_002645 and 99.3% identical to our laboratory strain. The r229E genome, compared with our lab strain, encodes amino acid differences in ORF1a (V416A, S2359R, T25124); ORF 4 a (D94Y); E protein (T36I); M protein (L82F); and spike protein (F230C, I700L). NL63 and 229E stocks were prepared in LLC-MK2 and HUH7 cells, respectively, as previously described (43, 45).

### Cell lines

Human A549 cells expressing the receptor ACE2 (A549^ACE2^) (16, 39) were cultured in RPMI 1640 (Gibco catalog no. 11875) supplemented with 10% fetal bovine serum (FBS) and 1× penicillin-streptomycin. LLC-MK2 cells were cultured in Minimum Essential Medium (MEM) α (Gibco 12571063) supplemented with 10% FBS and 1× of penicillin-streptomycin. MRC-5 (CCL-171) (77) and HT1080 (HT1080/CCL-121) (78) cells were cultured in Dulbecco’s modified Eagle’s medium (DMEM; Gibco 11965) supplemented with 10% FBS, 1× of penicillin-streptomycin. HUH7 cells were cultured in DMEM (Gibco 11965) supplemented with 10% FBS, 1× penicillin-streptomycin, 1% 100× MEM Non-Essential Amino Acids (NEAA; Gibco 11140050), and 1% GlutaMAX Supplement (Gibco 35050079).

### Primary nasal epithelial air-liquid interface (ALI) cultures

Nasal mucosal specimens were obtained via cytologic brushing of patients’ nares in the Department of Otorhinolaryngology-Head and Neck Surgery, Division of Rhinology at the University of Pennsylvania, and the Philadelphia Veteran Affairs Medical Center after obtaining informed consent. The full study protocol, including the acquisition and use of nasal specimens, was approved by the University of Pennsylvania Institutional Review Board (protocol #800614) and the Philadelphia VA Institutional Review Board (protocol #00781). Patients with a history of systemic disease or on immunosuppressive medications were excluded. ALI cultures were grown and differentiated on 0.4 µm pore transwell inserts as previously described (16, 44, 45). All nasal ALI cultures used in this study were differentiated after pooling nasal cells derived from four independent donors (in equal quantities) in order to limit donor-to-donor variability. ALI culture differentiation medium used for cultures was PneumaCult-ALI basal medium (Stemcell Technologies) (45).

### Viral replication kinetics and titration

For infection, immortalized cell lines were counted before seeding in 12-well plates (A549^ACE2^ and HT1080 cells at 3 × 10^5^ cells per well; MRC-5 cells at 5 × 10^5^ cells per well). The next day, supernatant samples containing virus were diluted in serum-free RPMI (A549^ACE2^ infections) or serum-free DMEM (MRC-5 infections) and added to cells for adsorption for 1 h at 33°C. After 1 h, cells were washed three times with PBS and fed with DMEM or RPMI supplemented with 2% FBS. For virus titration, 200 µL of the supernatant was collected at the times indicated and stored at −80°C for plaque assay on LLC-MK2 (NL63) or HUH7 (229E) cells as previously described (43). Nasal ALI cultures were apically infected at MOI = 5 PFU/cell with either NL63, 229E, r229E, or r229E-nsp15^mut^, apical surface liquid was collected via the addition of PBS, and viral titers were quantified via standard plaque assay as previously described (43, 44).

### Immunofluorescence (IF) staining for infected cells

Infections for IF staining to visualize infected cells (Fig. 2A and B) were conducted at an MOI of 1 using glass-bottom 12-well plates (Cellvis). Following infection, at indicated time points, cells were washed 3 times with 1× PBS and fixed in 4% paraformaldehyde at room temperature for 30 min. The cells were then washed 3 times with 1× PBS and permeabilized with 0.1% Triton X-100 in 1× PBS for 10 min and blocked with 2% Bovine Serum Albumin (BSA) in 1× PBS for 30 min at room temperature. Primary antibody incubation was done overnight at 4°C, followed by secondary incubation with Alexa Fluor dyes for 1 h at room temperature. See Spreadsheet S1 for the manufacturer and dilution used for each antibody. Images were acquired by immunofluorescence microscopy with a Nikon Eclipse Ti2 using a Nikon 20× Plan APO objective and Nikon DS-Qi1Mc-U3 12-bit camera. Images were processed using Fiji/ImageJ software.

### Intracellular nucleocapsid staining assay/flow cytometry

Briefly, indicated cells were cultured 24 h before infection at 33°C in T75 flasks. On the day of infection, the cells were infected with the indicated virus at an MOI of 1 or mock-infected and incubated for 1 h at 33°C. Following viral adsorption, the cells were washed, and fresh media was added. Cells were incubated until 24 or 48 hpi, at which time, the cells were harvested via trypsinization and washed twice with 1× PBS. Cells were then placed in 96-well U-bottom plates and were stained with LIVE/DEAD Fixable Aqua Dead Cell Stain at a 1:500 ratio (diluted in 1× PBS). Following initial staining, cells were fixed at 4°C for 30 min using the eBioscience fixation/permeabilization kit. After fixation and washing of cells, the cells were permeabilized using eBioscience Permeabilization Buffer (diluted to 1X using deionized water) for 5 min in the dark. Cells are then incubated with APC-conjugated (ab201807) NL63 or 229E Nucleocapsid antibodies at a ratio of 1:1,000 in 1× permeabilization buffer at 4°C overnight in the dark. The following day, the cells were washed, pelleted, and resuspended in 200 µL of 1× PBS. All samples were analyzed on a BD LSR-II analyzer and analyzed with FlowJo X software.

### Western blotting

Cell lysates were harvested at indicated time points using RIPA buffer (50 mM Tris pH 8, 150 mM NaCl, 0.5% deoxycholate, 0.1% SDS, 1% NP40) supplemented with protease inhibitors (Roche: cOmplete mini EDTA-free protease inhibitor) and phosphatase inhibitors (Roche: PhosStop easy pack). Lysates were harvested via scraping of the well or transwell insert and incubated on ice for 20 min, centrifuged for 20 min at 15,000 RPM at 4°C, and the supernatant was mixed 3:1 with 4× Laemmli sample buffer. Samples were boiled at 95°C for 5 min, then separated via sodium dodecyl sulfate-polyacrylamide gel electrophoresis (SDS/PAGE) and transferred to polyvinylidene difluoride (PVDF) membrane. Blots were blocked in 5% BSA or milk in 1X Tris-buffered saline with 0.1% Tween 20 Detergent (TBST) and probed with antibodies as listed in Spreadsheet S1. Blots were visualized using Thermo Scientific SuperSignal West Femto Substrate. Blots were stripped using Thermo Scientific Restore Western Blot stripping buffer for 1 h at room temperature and then re-blocked and probed sequentially with antibodies (54).

### Quantitative PCR (q-PCR)

Cells were lysed at the indicated time points with buffer RLT Plus (Qiagen RNeasy Plus Kit #74106), and total RNA was extracted following the manufacturer’s protocol. RNA was reverse transcribed into complementary DNA (cDNA) using the High-Capacity Reverse Transcriptase Kit (Applied Biosystems). This cDNA was amplified using specific qRT-PCR primers for each target gene, iQ SYBR Green Supermix (Bio-Rad), and the QuantStudio 3 PCR system (Thermo Fisher). See Spreadsheet S1 for primer sequences used for each target. Technical triplicates were averaged, and changes in mRNA levels were reported as fold change over mock, using the formula 2^-Δ(ΔCt)^. ΔCt values were calculated using the formula ΔCt = Ct_gene of interest_ – Ct_18S_. Δ(ΔCt) was calculated by subtracting mock-infected ΔCt values from ΔCt values for NL63- or 229E-infected samples (54).

### rRNA degradation assay

Total RNA was harvested with buffer RLT Plus (Qiagen RNeasy Plus Kit #74106) and analyzed on an RNA chip with an Agilent Bioanalyzer using the Agilent 196 RNA 6000 Nano Kit and its prescribed protocol as we have described previously (16).

### Immunofluorescence (IF) assay for dsRNA and PABPC1 quantification

The indicated cell type was seeded onto glass coverslips (Thomas Scientific: 1203J81) and infected as described above. At the indicated time point, samples were fixed with 4% paraformaldehyde (PFA) for 30 min prior followed by permeabilization in 70% ethanol. For IFA, the samples were incubated with the indicated primary antibody diluted in 1× PBS at 4°C overnight (Spreadsheet S1). Secondary antibodies were Alexa Fluor antibodies purchased from Abcam and added at 1:1,000 in 1× PBS. Cells were washed 3 times in 1× PBS, then secondary antibodies were diluted in 1× PBS and added for 2 h at room temperature. Coverslips were then washed 3 times in 1× PBS before being mounted on coverslides (Fisher: 12-544-11) with Vectashield (Vector Laboratories: 101098-044) or ProLong Gold Antifade Mountant (Invitrogen: P36931). Images were taken on a Nikon Eclipse Ti2 with a CFI60 Plan Apochromat Lambda D 100 x Oil Immersion Objective Lens, N.A. 1.45, W.D. 0.13 mm, F.O.V. 25 mm, DIC, Spring Loaded. The filter set included: C-FL DAPI Filter Set, High-Signal-Noise, Semrock Brightline, Excitation: 356/30 nm (341-371nm). Image processing and analysis were performed using FIJI 2.16.0 (ImageJ2), and data processing was conducted in Microsoft Excel.

### smRNA-FISH for quantification of GAPDH degradation

For smFISH, the cells were seeded onto glass coverslips and infected as described for IF. After 4% PFA fixation and permeabilization of 70% EtOH, coverslips were placed in buffer A (filter-sterilized 2× SSC with 10% formamide) for 5 min. For each sample, smFISH probes (sequences provided in Spreadsheet S1) were prepared as described in (51) and added 1:100 into 50 μL of hybridization buffer: 10% dextran sulfate (Fisher Scientific Co LLC: S4030), 10% formamide (Fisher Scientific Co LLC: BP227500), and 1× nuclease-free SSC (Life Technologies Corporation: 15557044). The hybridization buffer containing the probes was added onto parafilm placed inside a Petri dish (Fisher: FB0875711A). The coverslips were then incubated with the smFISH probes overnight at 37°C. The following day, coverslips were washed, mounted on slides, and imaged.

### Statistical analysis

Plotting of data and statistical analysis were performed using GraphPad Prism software (GraphPad Software, Inc.). Statistical significance was determined by comparing mutant virus (r229E-nsp15^mut^) to WT 229E (r229E) using repeated measures two-way ANOVA for viral replication curves and qRT-PCR. Displayed significance is determined by *P* value, where * =*P* < 0.05; ** =*P* < 0.01; *** =*P* < 0.001; and **** =*P* < 0.0001; ns = not significant, ns is not displayed on the graph.

## Data Availability

The genome RNA of our laboratory strains, NL63 and 229E, was sequenced and submitted to GenBank under accession numbers PX640308 and PX640306, respectively. Upon request, all data are fully available and without restriction.

## AFTER EPUB

*[This article was published on 10 December 2025 with an incorrect accession number in the Data Availability statement. The number was updated in the current version, posted on 17 December 2025.]*

## SUPPLEMENTAL MATERIAL

Spreadsheet S1 mbio.03203-25-s0001.xlsx Antibodies, smFISH probes, and qPCR primers.

Supplemental figures mbio.03203-25-s0002.pdf Fig. S1 to S4.

Supplemental legends mbio.03203-25-s0003.pdf Legends for Fig. S1 to S4.

ASM does not own the copyrights to Supplemental Material that may be linked to, or accessed through, an article. The authors have granted ASM a non-exclusive, world-wide license to publish the Supplemental Material files. Please contact the corresponding author directly for reuse.

## References

[1] Weiss SR, Leibowitz JL. 2011. Coronavirus pathogenesis. Adv Virus Res 81:85–164. doi:10.1016/B978-0-12-385885-6.00009-2 22094080 PMC7149603

[2] Weiss SR. 2020. Forty years with coronaviruses. J Exp Med 217:e20200537. doi:10.1084/jem.20200537 32232339 PMC7103766

[3] V’kovski P, Kratzel A, Steiner S, Stalder H, Thiel V. 2021. Coronavirus biology and replication: implications for SARS-CoV-2. Nat Rev Microbiol 19:155–170. doi:10.1038/s41579-020-00468-6 33116300 PMC7592455

[4] Perlman S, Netland J. 2009. Coronaviruses post-SARS: update on replication and pathogenesis. Nat Rev Microbiol 7:439–450. doi:10.1038/nrmicro2147 19430490 PMC2830095

[5] Liu DX, Liang JQ, Fung TS. 2021. Human coronavirus-229E, -OC43, -NL63, and -HKU1 (Coronaviridae), p 428–440. In Encyclopedia of virology. Elsevier.

[6] McIntosh K, Dees JH, Becker WB, Kapikian AZ, Chanock RM. 1967. Recovery in tracheal organ cultures of novel viruses from patients with respiratory disease. Proc Natl Acad Sci USA 57:933–940. doi:10.1073/pnas.57.4.933 5231356 PMC224637

[7] van der Hoek L, Pyrc K, Berkhout B. 2006. Human coronavirus NL63, a new respiratory virus. FEMS Microbiol Rev 30:760–773. doi:10.1111/j.1574-6976.2006.00032.x 16911043 PMC7109777

[8] Corman VM, Muth D, Niemeyer D, Drosten C. 2018. Hosts and sources of endemic human coronaviruses. Adv Virus Res 100:163–188. doi:10.1016/bs.aivir.2018.01.001 29551135 PMC7112090

[9] Gorse GJ, O’Connor TZ, Hall SL, Vitale JN, Nichol KL. 2009. Human coronavirus and acute respiratory illness in older adults with chronic obstructive pulmonary disease. J Infect Dis 199:847–857. doi:10.1086/597122 19239338 PMC7110218

[10] Principi N, Bosis S, Esposito S. 2010. Effects of coronavirus infections in children. Emerg Infect Dis 16:183–188. doi:10.3201/eid1602.090469 20113545 PMC2957994

[11] Fielding BC. 2011. Human coronavirus NL63: a clinically important virus? Future Microbiol 6:153–159. doi:10.2217/fmb.10.166 21366416 PMC7079714

[12] Mayer K, Nellessen C, Hahn‐Ast C, Schumacher M, Pietzonka S, Eis‐Hübinger AM, Drosten C, Brossart P, Wolf D. 2016. Fatal outcome of human coronavirus NL63 infection despite successful viral elimination by IFN-alpha in a patient with newly diagnosed ALL. Eur J Haematol 97:208–210. doi:10.1111/ejh.12744 26854965 PMC7163643

[13] Nunes MC, Kuschner Z, Rabede Z, Madimabe R, Van Niekerk N, Moloi J, Kuwanda L, Rossen JW, Klugman KP, Adrian PV, Madhi SA. 2014. Clinical epidemiology of bocavirus, rhinovirus, two polyomaviruses and four coronaviruses in HIV-infected and HIV-uninfected South African children. PLoS One 9:e86448. doi:10.1371/journal.pone.0086448 24498274 PMC3911925

[14] Liu DX, Fung TS, Chong KK-L, Shukla A, Hilgenfeld R. 2014. Accessory proteins of SARS-CoV and other coronaviruses. Antiviral Res 109:97–109. doi:10.1016/j.antiviral.2014.06.013 24995382 PMC7113789

[15] Fung TS, Liu DX. 2019. Human coronavirus: host-pathogen interaction. Annu Rev Microbiol 73:529–557. doi:10.1146/annurev-micro-020518-115759 31226023

[16] Li Y, Renner DM, Comar CE, Whelan JN, Reyes HM, Cardenas-Diaz FL, Truitt R, Tan LH, Dong B, Alysandratos KD, Huang J, Palmer JN, Adappa ND, Kohanski MA, Kotton DN, Silverman RH, Yang W, Morrisey EE, Cohen NA, Weiss SR. 2021. SARS-CoV-2 induces double-stranded RNA-mediated innate immune responses in respiratory epithelial-derived cells and cardiomyocytes. Proc Natl Acad Sci USA 118. doi:10.1073/pnas.2022643118 PMC8072330 33811184

[17] Tanneti NS, Stillwell HA, Weiss SR. 2025. Human coronaviruses: activation and antagonism of innate immune responses. Microbiol Mol Biol Rev 89:e0001623. doi:10.1128/mmbr.00016-23 39699237 PMC11948496

[18] Roth-Cross JK, Bender SJ, Weiss SR. 2008. Murine coronavirus mouse hepatitis virus is recognized by MDA5 and induces type I interferon in brain macrophages/microglia. J Virol 82:9829–9838. doi:10.1128/JVI.01199-08 18667505 PMC2566260

[19] Hur S. 2019. Double-stranded RNA sensors and modulators in innate immunity. Annu Rev Immunol 37:349–375. doi:10.1146/annurev-immunol-042718-041356 30673536 PMC7136661

[20] Boncristiani HF, Criado MF, Arruda E. 2009. Respiratory viruses, p 500–518. In Encyclopedia of microbiology

[21] Hariri BM, Cohen NA. 2016. New insights into upper airway innate immunity. Am J Rhinol Allergy 30:319–323. doi:10.2500/ajra.2016.30.4360 27657896 PMC5013235

[22] Vareille M, Kieninger E, Edwards MR, Regamey N. 2011. The airway epithelium: soldier in the fight against respiratory viruses. Clin Microbiol Rev 24:210–229. doi:10.1128/CMR.00014-10 21233513 PMC3021210

[23] Parker D, Prince A. 2011. Innate immunity in the respiratory epithelium. Am J Respir Cell Mol Biol 45:189–201. doi:10.1165/rcmb.2011-0011RT 21330463 PMC3175551

[24] Kikkert M. 2020. Innate immune evasion by human respiratory RNA viruses. J Innate Immun 12:4–20. doi:10.1159/000503030 31610541 PMC6959104

[25] Gantier MP, Williams BRG. 2007. The response of mammalian cells to double-stranded RNA. Cytokine Growth Factor Rev 18:363–371. doi:10.1016/j.cytogfr.2007.06.016 17698400 PMC2084215

[26] Silverman RH. 2007. Viral encounters with 2′,5′-oligoadenylate synthetase and RNase L during the interferon antiviral response. J Virol 81:12720–12729. doi:10.1128/JVI.01471-07 17804500 PMC2169107

[27] Ulferts R, Ziebuhr J. 2011. Nidovirus ribonucleases: structures and functions in viral replication. RNA Biol 8:295–304. doi:10.4161/rna.8.2.15196 21422822

[28] Otter CJ, Bracci N, Parenti NA, Ye C, Tan LH, Asthana A, Pfannenstiel JJ, Jackson N, Fehr AR, Silverman RH, Cohen NA, Martinez-Sobrido L, Weiss SR. 2023. SARS-CoV-2 nsp15 endoribonuclease antagonizes dsRNA-induced antiviral signaling. bioRxiv:2023.11.15.566945. doi:10.1101/2023.11.15.566945 PMC11009620 38568967

[29] Comar CE, Otter CJ, Pfannenstiel J, Doerger E, Renner DM, Tan LH, Perlman S, Cohen NA, Fehr AR, Weiss SR. 2022. MERS-CoV endoribonuclease and accessory proteins jointly evade host innate immunity during infection of lung and nasal epithelial cells. Proc Natl Acad Sci USA 119:e2123208119. doi:10.1073/pnas.2123208119 35594398 PMC9173776

[30] Deng X, Baker SC. 2018. An “Old” protein with a new story: coronavirus endoribonuclease is important for evading host antiviral defenses. Virology (Auckl) 517:157–163. doi:10.1016/j.virol.2017.12.024 PMC5869138 29307596

[31] Ancar R, Li Y, Kindler E, Cooper DA, Ransom M, Thiel V, Weiss SR, Hesselberth JR, Barton DJ. 2020. Physiologic RNA targets and refined sequence specificity of coronavirus EndoU. RNA 26:1976–1999. doi:10.1261/rna.076604.120 32989044 PMC7668261

[32] Kindler E, Gil-Cruz C, Spanier J, Li Y, Wilhelm J, Rabouw HH, Züst R, Hwang M, V’kovski P, Stalder H, Marti S, Habjan M, Cervantes-Barragan L, Elliot R, Karl N, Gaughan C, van Kuppeveld FJM, Silverman RH, Keller M, Ludewig B, Bergmann CC, Ziebuhr J, Weiss SR, Kalinke U, Thiel V. 2017. Early endonuclease-mediated evasion of RNA sensing ensures efficient coronavirus replication. PLoS Pathog 13:e1006195. doi:10.1371/journal.ppat.1006195 28158275 PMC5310923

[33] Hackbart M, Deng X, Baker SC. 2020. Coronavirus endoribonuclease targets viral polyuridine sequences to evade activating host sensors. Proc Natl Acad Sci USA 117:8094–8103. doi:10.1073/pnas.1921485117 32198201 PMC7149396

[34] Deng X, van Geelen A, Buckley AC, O’Brien A, Pillatzki A, Lager KM, Faaberg KS, Baker SC. 2019. Coronavirus endoribonuclease activity in porcine epidemic diarrhea virus suppresses type I and type III interferon responses. J Virol 93:e02000-18. doi:10.1128/JVI.02000-18 30728254 PMC6450110

[35] Gao B, Gong X, Fang S, Weng W, Wang H, Chu H, Sun Y, Meng C, Tan L, Song C, Qiu X, Liu W, Forlenza M, Ding C, Liao Y. 2021. Inhibition of anti-viral stress granule formation by coronavirus endoribonuclease nsp15 ensures efficient virus replication. PLoS Pathog 17:e1008690. doi:10.1371/journal.ppat.1008690 33635931 PMC7946191

[36] Deng X, Buckley AC, Pillatzki A, Lager KM, Baker SC, Faaberg KS. 2021. Development and utilization of an infectious clone for porcine deltacoronavirus strain USA/IL/2014/026. Virology (Auckl) 553:35–45. doi:10.1016/j.virol.2020.11.002 PMC7664480 33220618

[37] Kindler E, Thiel V. 2014. To sense or not to sense viral RNA — essentials of coronavirus innate immune evasion. Curr Opin Microbiol 20:69–75. doi:10.1016/j.mib.2014.05.005 24908561 PMC7108419

[38] Otter CJ, Bracci N, Parenti NA, Ye C, Asthana A, Blomqvist EK, Tan LH, Pfannenstiel JJ, Jackson N, Fehr AR, Silverman RH, Burke JM, Cohen NA, Martinez-Sobrido L, Weiss SR. 2024. SARS-CoV-2 nsp15 endoribonuclease antagonizes dsRNA-induced antiviral signaling. Proc Natl Acad Sci USA 121:e2320194121. doi:10.1073/pnas.2320194121 38568967 PMC11009620

[39] Giard DJ, Aaronson SA, Todaro GJ, Arnstein P, Kersey JH, Dosik H, Parks WP. 1973. In vitro cultivation of human tumors: establishment of cell lines derived from a series of solid tumors. J Natl Cancer Inst 51:1417–1423. doi:10.1093/jnci/51.5.1417 4357758

[40] Hofmann H, Pyrc K, van der Hoek L, Geier M, Berkhout B, Pöhlmann S. 2005. Human coronavirus NL63 employs the severe acute respiratory syndrome coronavirus receptor for cellular entry. Proc Natl Acad Sci USA 102:7988–7993. doi:10.1073/pnas.0409465102 15897467 PMC1142358

[41] Li W, Sui J, Huang IC, Kuhn JH, Radoshitzky SR, Marasco WA, Choe H, Farzan M. 2007. The S proteins of human coronavirus NL63 and severe acute respiratory syndrome coronavirus bind overlapping regions of ACE2. Virology (Auckl) 367:367–374. doi:10.1016/j.virol.2007.04.035 PMC2693060 17631932

[42] Otter CJ, Renner DM, Fausto A, Tan LH, Cohen NA, Weiss SR. 2024. Interferon signaling in the nasal epithelium distinguishes among lethal and common cold coronaviruses and mediates viral clearance. Proc Natl Acad Sci USA 121:e2402540121. doi:10.1073/pnas.2402540121 38758698 PMC11127059

[43] Fausto A, Otter CJ, Bracci N, Weiss SR. 2023. Improved culture methods for human coronaviruses HCoV-OC43, HCoV-229E, and HCoV-NL63. Current Protocols 3:e914. doi:10.1002/cpz1.914 37882768 PMC10695105

[44] Otter CJ, Fausto A, Tan LH, Weiss SR, Cohen NA. 2023. Infection of primary nasal epithelial cells grown at an air-liquid interface to characterize human coronavirus-host interactions. J Vis Exp. doi:10.3791/64868 PMC10811614 37811957

[45] Otter CJ, Fausto A, Tan LH, Khosla AS, Cohen NA, Weiss SR. 2023. Infection of primary nasal epithelial cells differentiates among lethal and seasonal human coronaviruses. Proc Natl Acad Sci USA 120:e2218083120. doi:10.1073/pnas.2218083120 37023127 PMC10104492

[46] Otter CJ, Renner DM, Fausto A, Tan LH, Cohen NA, Weiss SR. 2023. Interferon signaling in the nasal epithelium distinguishes among lethal and common cold respiratory viruses and is critical for viral clearance. bioRxiv. doi:10.1101/2023.12.18.571720 PMC11127059 38758698

[47] Pyrc K, Dijkman R, Deng L, Jebbink MF, Ross HA, Berkhout B, van der Hoek L. 2006. Mosaic structure of human coronavirus NL63, one thousand years of evolution. J Mol Biol 364:964–973. doi:10.1016/j.jmb.2006.09.074 17054987 PMC7094706

[48] Whelan JN, Parenti NA, Hatterschide J, Renner DM, Li Y, Reyes HM, Dong B, Perez ER, Silverman RH, Weiss SR. 2021. Zika virus employs the host antiviral RNase L protein to support replication factory assembly. Proc Natl Acad Sci USA 118:e2101713118. doi:10.1073/pnas.2101713118 34031250 PMC8179202

[49] Whelan JN, Li Y, Silverman RH, Weiss SR. 2019. Zika virus production is resistant to RNase L antiviral activity. J Virol 93:e00313-19. doi:10.1128/JVI.00313-19 31142667 PMC6675901

[50] Eriksson KK, Makia D, Thiel V. 2008. Generation of recombinant coronaviruses using vaccinia virus as the cloning vector and stable cell lines containing coronaviral replicon RNAs. Methods Mol Biol 454:237–254. doi:10.1007/978-1-59745-181-9_18 19057873 PMC7121376

[51] Cusic R, Watkins JM, Burke JM. 2023. Single-cell analysis of RNase L-mediated mRNA decay. Methods Enzymol 692:157–175. doi:10.1016/bs.mie.2023.04.016 37925178

[52] Burke JM, Ripin N, Ferretti MB, St Clair LA, Worden-Sapper ER, Salgado F, Sawyer SL, Perera R, Lynch KW, Parker R. 2022. RNase L activation in the cytoplasm induces aberrant processing of mRNAs in the nucleus. PLoS Pathog 18:e1010930. doi:10.1371/journal.ppat.1010930 36318584 PMC9651596

[53] Burke JM, Moon SL, Matheny T, Parker R. 2019. RNase L reprograms translation by widespread mRNA turnover escaped by antiviral mRNAs. Mol Cell 75:1203–1217. doi:10.1016/j.molcel.2019.07.029 31494035 PMC6754297

[54] Comar CE, Goldstein SA, Li Y, Yount B, Baric RS, Weiss SR. 2019. Antagonism of dsRNA-induced innate immune pathways by NS4a and NS4b accessory proteins during MERS coronavirus infection. mBio 10:e00319-19. doi:10.1128/mBio.00319-19 30914508 PMC6437052

[55] Thornbrough JM, Jha BK, Yount B, Goldstein SA, Li Y, Elliott R, Sims AC, Baric RS, Silverman RH, Weiss SR. 2016. Middle East respiratory syndrome coronavirus NS4b protein inhibits host RNase L activation. mBio 7:e00258. doi:10.1128/mBio.00258-16 27025250 PMC4817253

[56] Fung S-Y, Yuen K-S, Ye Z-W, Chan C-P, Jin D-Y. 2020. A tug-of-war between severe acute respiratory syndrome coronavirus 2 and host antiviral defence: lessons from other pathogenic viruses. Emerg Microbes Infect 9:558–570. doi:10.1080/22221751.2020.1736644 32172672 PMC7103735

[57] Sievers BL, Cheng MTK, Csiba K, Meng B, Gupta RK. 2024. SARS-CoV-2 and innate immunity: the good, the bad, and the “goldilocks”. Cell Mol Immunol 21:171–183. doi:10.1038/s41423-023-01104-y 37985854 PMC10805730

[58] Li Z, Tomlinson ACA, Wong AHM, Zhou D, Desforges M, Talbot PJ, Benlekbir S, Rubinstein JL, Rini JM. 2019. The human coronavirus HCoV-229E S-protein structure and receptor binding. eLife 8:1–22. doi:10.7554/eLife.51230 PMC6970540 31650956

[59] Yeager CL, Ashmun RA, Williams RK, Cardellichio CB, Shapiro LH, Look AT, Holmes KV. 1992. Human aminopeptidase N is a receptor for human coronavirus 229E. Nature 357:420–422. doi:10.1038/357420a0 1350662 PMC7095410

[60] Milewska A, Nowak P, Owczarek K, Szczepanski A, Zarebski M, Hoang A, Berniak K, Wojarski J, Zeglen S, Baster Z, Rajfur Z, Pyrc K. 2018. Entry of human coronavirus NL63 into the cell. J Virol 92:e01933-17. doi:10.1128/JVI.01933-17 29142129 PMC5774871

[61] Bender SJ, Phillips JM, Scott EP, Weiss SR. 2010. Murine coronavirus receptors are differentially expressed in the central nervous system and play virus strain-dependent roles in neuronal spread. J Virol 84:11030–11044. doi:10.1128/JVI.02688-09 20739537 PMC2953140

[62] Lim S, Zhang M, Chang TL. 2022. ACE2-independent alternative receptors for SARS-CoV-2. Viruses 14:2535. doi:10.3390/v14112535 36423144 PMC9692829

[63] Duncan JKS, Xu D, Licursi M, Joyce MA, Saffran HA, Liu K, Gohda J, Tyrrell DL, Kawaguchi Y, Hirasawa K. 2023. Interferon regulatory factor 3 mediates effective antiviral responses to human coronavirus 229E and OC43 infection. Front Immunol 14:930086. doi:10.3389/fimmu.2023.930086 37197656 PMC10183588

[64] Loo S-L, Wark P, Esneau C, Nichol KS, Hsu ACY, Bartlett NW. 2020. Human coronaviruses 229E and OC43 replicate and induce distinct antiviral responses in differentiated primary human bronchial epithelial cells. Am J Physiol Lung Cell Mol Physiol 319:L926–L931. doi:10.1152/ajplung.00374.2020 32903043 PMC7758816

[65] Pakos-Zebrucka K, Koryga I, Mnich K, Ljujic M, Samali A, Gorman AM. 2016. The integrated stress response. EMBO Rep 17:1374–1395. doi:10.15252/embr.201642195 27629041 PMC5048378

[66] Wek RC. 2018. Role of eIF2α kinases in translational control and adaptation to cellular stress. Cold Spring Harb Perspect Biol 10:a032870. doi:10.1101/cshperspect.a032870 29440070 PMC6028073

[67] Renner DM, Parenti NA, Bracci N, Weiss SR. 2025. Betacoronaviruses differentially activate the integrated stress response to optimize viral replication in lung-derived cell lines. Viruses 17:120. doi:10.3390/v17010120 39861909 PMC11769277

[68] Deng X, Hackbart M, Mettelman RC, O’Brien A, Mielech AM, Yi G, Kao CC, Baker SC. 2017. Coronavirus nonstructural protein 15 mediates evasion of dsRNA sensors and limits apoptosis in macrophages. Proc Natl Acad Sci USA 114:E4251–E4260. doi:10.1073/pnas.1618310114 28484023 PMC5448190

[69] Birdwell LD, Zalinger ZB, Li Y, Wright PW, Elliott R, Rose KM, Silverman RH, Weiss SR. 2016. Activation of RNase L by murine coronavirus in myeloid cells is dependent on basal Oas gene expression and independent of virus-induced interferon. J Virol 90:3160–3172. doi:10.1128/JVI.03036-15 26739051 PMC4810646

[70] Zhao L, Birdwell LD, Wu A, Elliott R, Rose KM, Phillips JM, Li Y, Grinspan J, Silverman RH, Weiss SR. 2013. Cell-type-specific activation of the oligoadenylate synthetase-RNase L pathway by a murine coronavirus. J Virol 87:8408–8418. doi:10.1128/JVI.00769-13 23698313 PMC3719824

[71] Li Y, Weiss SR. 2016. Antagonism of RNase L is required for murine coronavirus replication in Kupffer cells and liver sinusoidal endothelial cells but not in hepatocytes. J Virol 90:9826–9832. doi:10.1128/JVI.01423-16 27558415 PMC5068532

[72] Narayanan K, Huang C, Makino S. 2008. SARS coronavirus accessory proteins. Virus Res 133:113–121. doi:10.1016/j.virusres.2007.10.009 18045721 PMC2720074

[73] Müller MA, van der Hoek L, Voss D, Bader O, Lehmann D, Schulz AR, Kallies S, Suliman T, Fielding BC, Drosten C, Niedrig M. 2010. Human coronavirus NL63 open reading frame 3 encodes a virion-incorporated N-glycosylated membrane protein. Virol J 7:6. doi:10.1186/1743-422X-7-6 20078868 PMC2819038

[74] Dijkman R, Jebbink MF, Wilbrink B, Pyrc K, Zaaijer HL, Minor PD, Franklin S, Berkhout B, Thiel V, van der Hoek L. 2006. Human coronavirus 229E encodes a single ORF4 protein between the spike and the envelope genes. Virol J 3:106. doi:10.1186/1743-422X-3-106 17194306 PMC1774570

[75] Zhang R, Wang K, Lv W, Yu W, Xie S, Xu K, Schwarz W, Xiong S, Sun B. 2014. The ORF4a protein of human coronavirus 229E functions as a viroporin that regulates viral production. Biochim Biophys Acta 1838:1088–1095. doi:10.1016/j.bbamem.2013.07.025 23906728 PMC7094429

[76] Thiel V, Herold J, Schelle B, Siddell SG. 2001. Infectious RNA transcribed in vitro from a cDNA copy of the human coronavirus genome cloned in vaccinia virus. J Gen Virol 82:1273–1281. doi:10.1099/0022-1317-82-6-1273 11369870

[77] Jacobs JP, Jones CM, Baille JP. 1970. Characteristics of a human diploid cell designated MRC-5. Nature 227:168–170. doi:10.1038/227168a0 4316953

[78] Rasheed S, Nelson-Rees WA, Toth EM, Arnstein P, Gardner MB. 1974. Characterization of a newly derived human sarcoma cell line (HT-1080). Cancer 33:1027–1033. doi:10.1002/1097-0142(197404)33:4<1027::aid-cncr2820330419>3.0.co;2-z 4132053

