# Supplementary figures and images for: Human alphacoronavirus replication and innate immune induction in airway culture systems
Source: mBio. 2025 Dec 10;17(1):e03203-25. doi: 10.1128/mbio.03203-25 (PMC12802182; doi:10.1128/mbio.03203-25)

Figure S1

# Gating Strategy

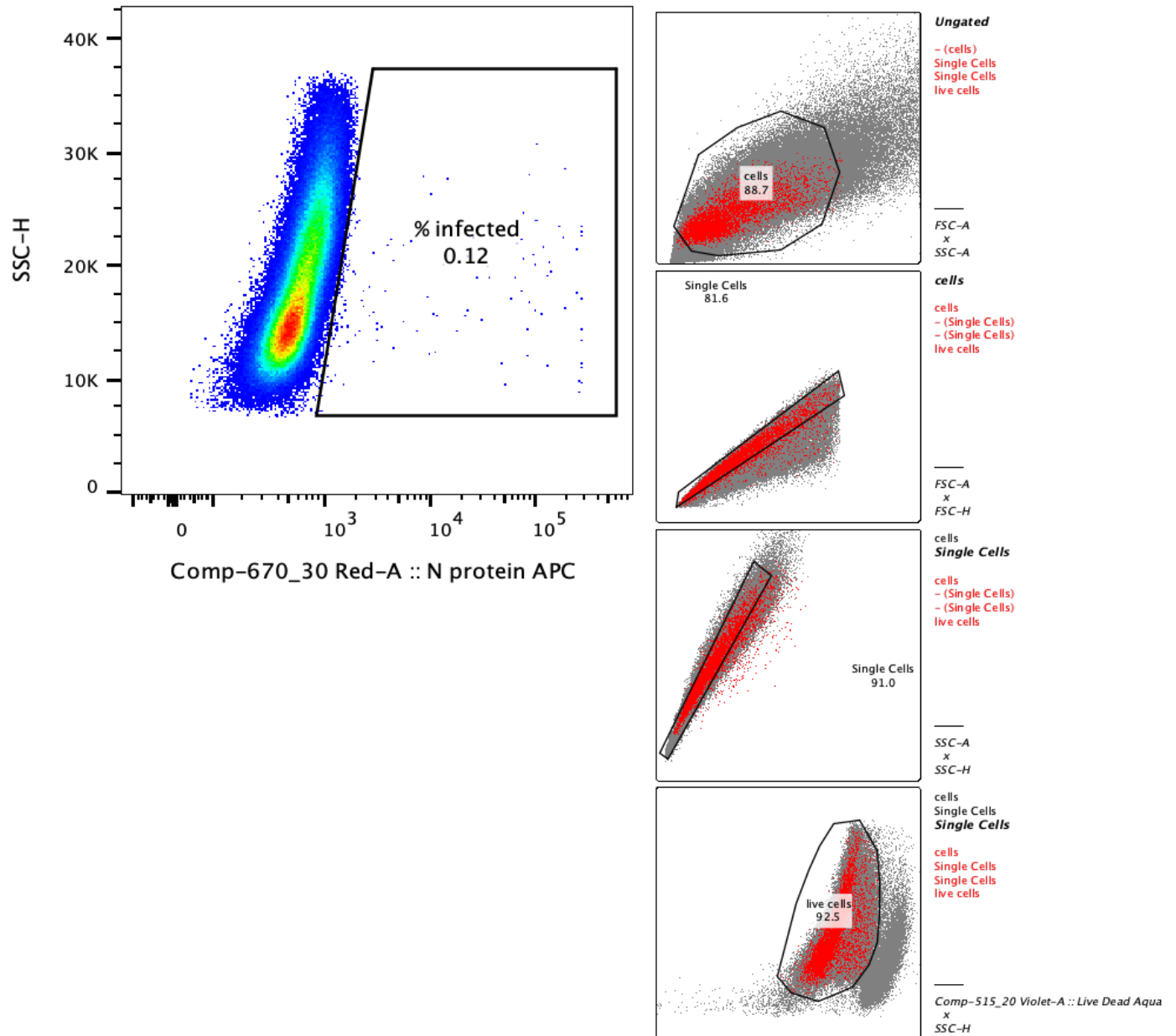

Fig S2

A549<sup>ACE2</sup>

MRC-5

Nasal ALI

ACE2

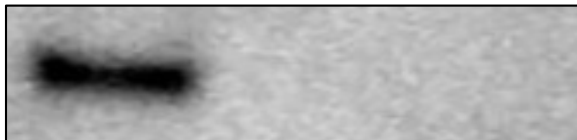

APN

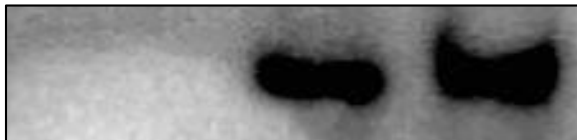

GAPDH

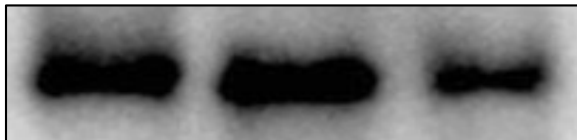

Figure S3

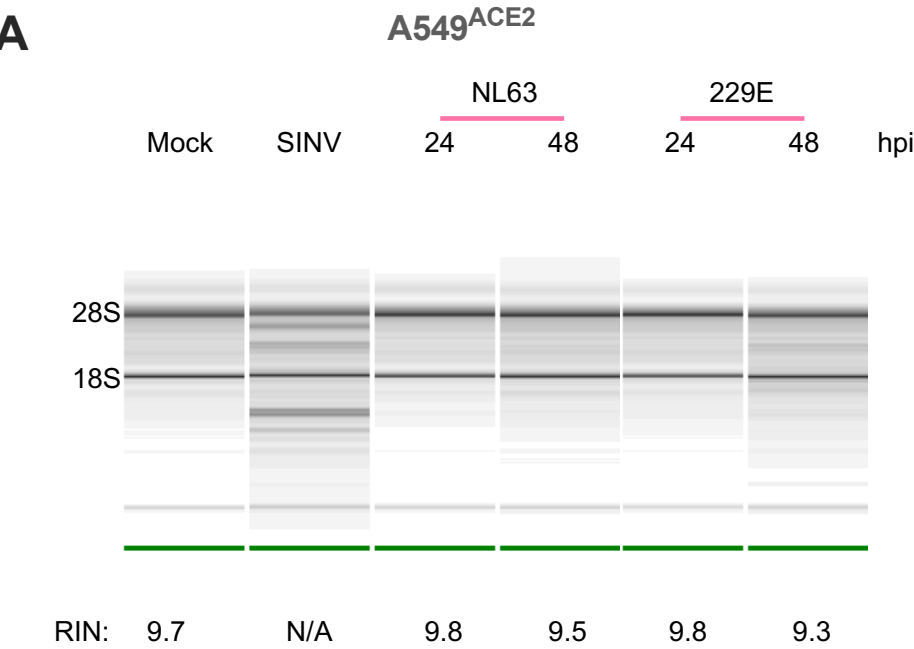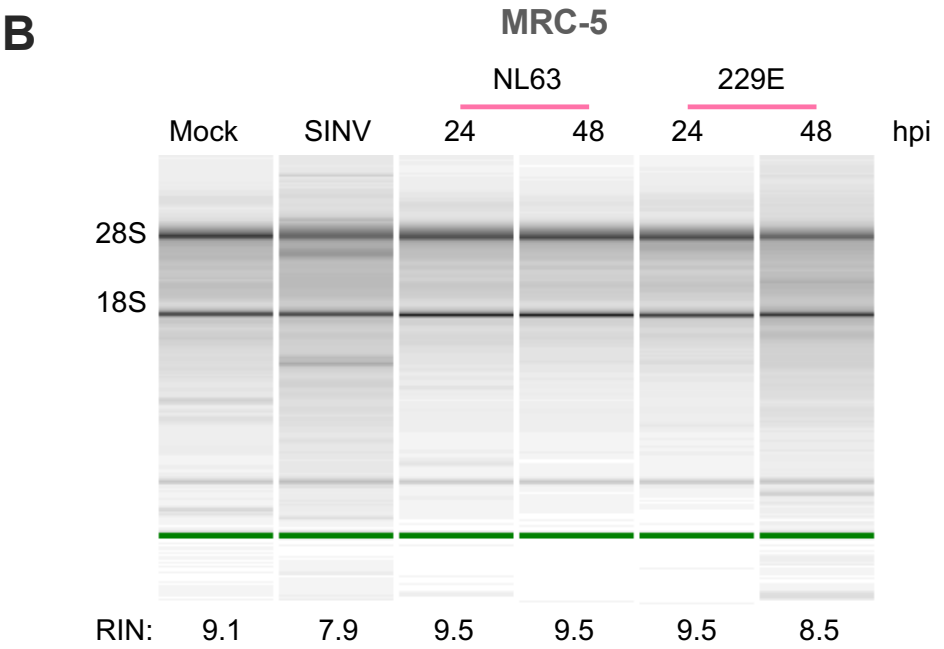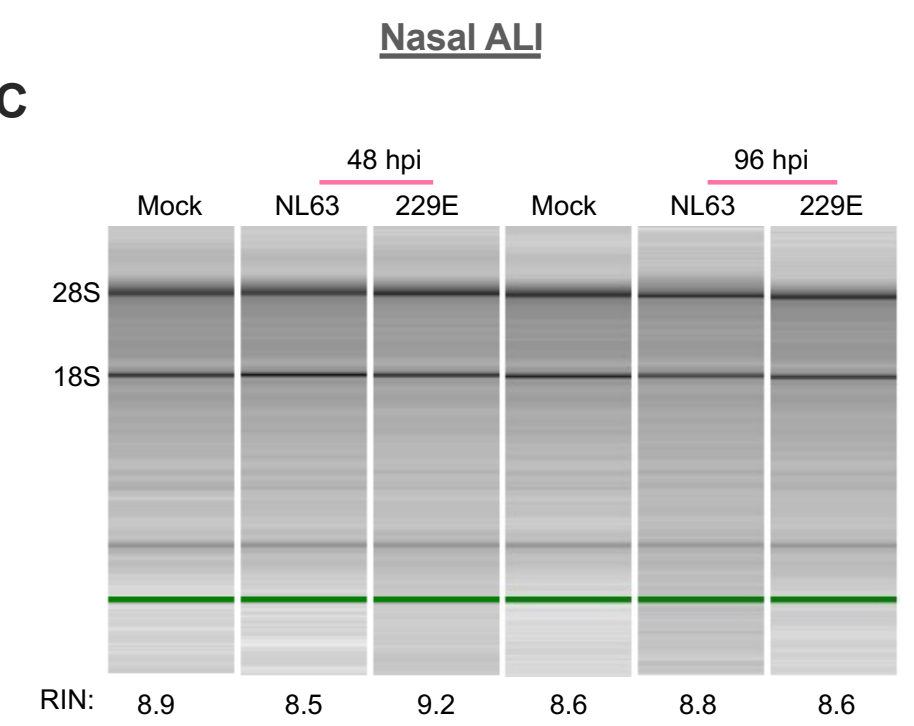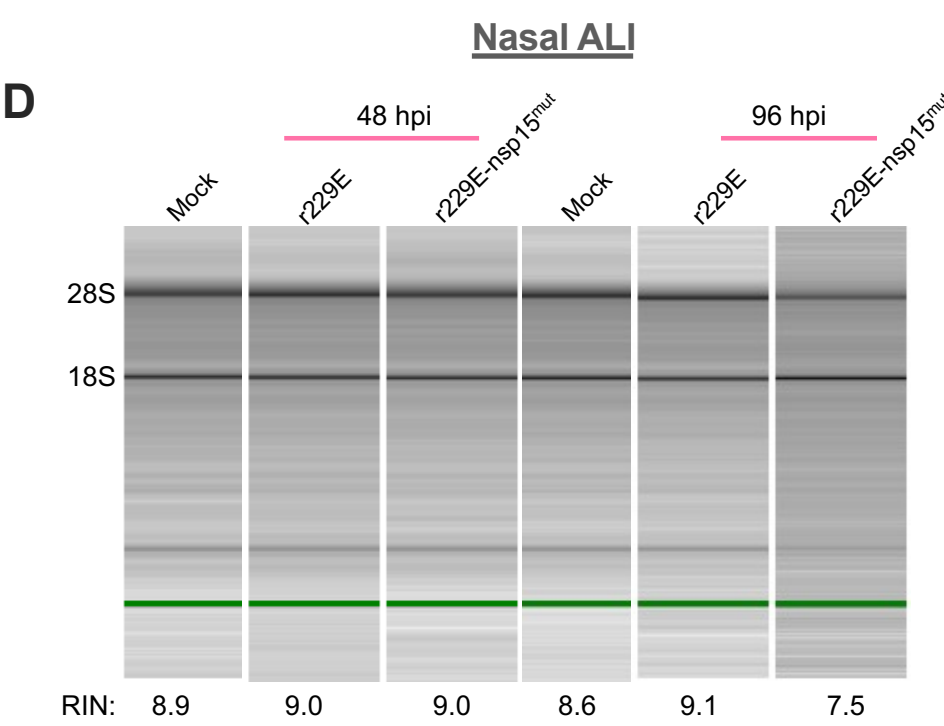

# Figure S4

## A

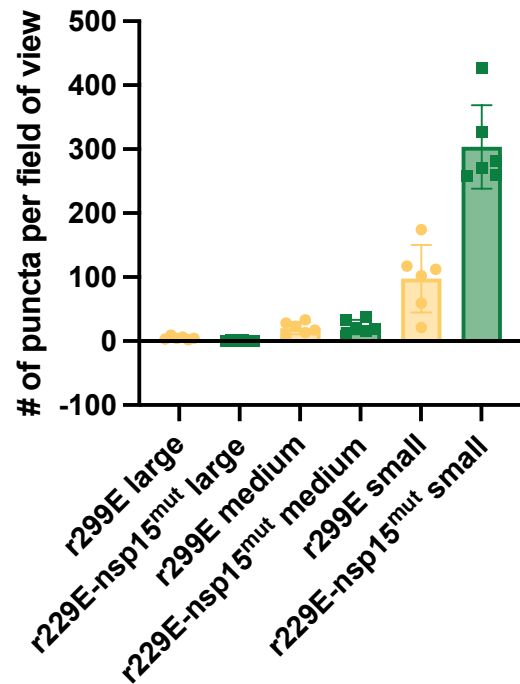

## B

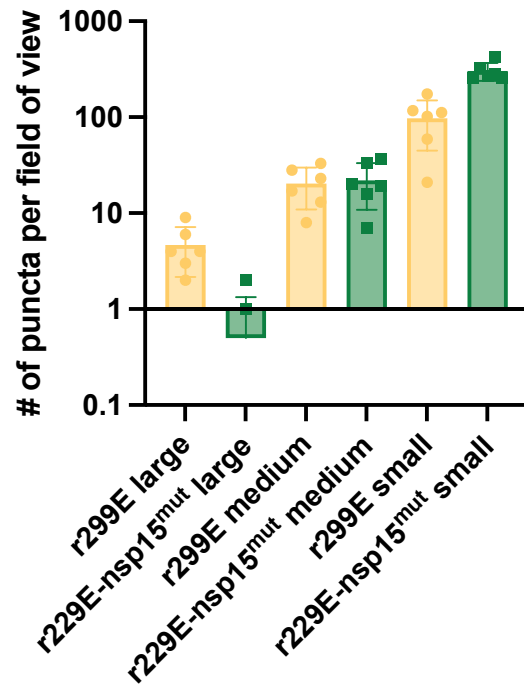

Supplement: Supplemental figures — Fig. S1 to S4. [file mbio.03203-25-s0002.pdf]
