# Supplementary material for: Human alphacoronavirus replication and innate immune induction in airway culture systems
Source: mBio. 2025 Dec 10;17(1):e03203-25. doi: 10.1128/mbio.03203-25 (PMC12802182; doi:10.1128/mbio.03203-25)
Supplement: Supplemental legends — Legends for Fig. S1 to S4. [file mbio.03203-25-s0003.pdf]

Figure S1. Gating strategy for determining percent infected cells. A representative gating strategy used to identify the percent infected cells in indicated cell populations is shown above.

Figure S2. Expression of NL63 and 229E receptors. Mock-infected A549^{ACE2,} MRC-5, and nasal ALI cultures were analyzed via western blot for expression of virus receptors. Cell lysates were harvested and proteins were separated by SDS/PAGE and immunoblotted with antibodies against ACE2, APN and GAPDH. Data are from one representative of two independent experiments.

Figure S3. NL63 and 229E do not activate the OAS/RNase L pathway. (A-C) Indicated cell type was mock-infected or infected in triplicate at MOI = 5 with NL63 or 229E. Total cellular RNA was harvested at indicated times post-infection. The degree of rRNA degradation was assessed on an Agilent Bioanalyzer. 28S and 18S rRNA positions are indicated. RNA integrity numbers (RIN) are shown under each lane, with lower RIN indicating increased RNA degradation. RIN for SINV positive control sample is N/A, which often occurs when rRNA degradation is very extensive. Data are from one representative of two independent experiments.

Figure S4 229E-nsp15^{mut} infection results in a larger number of small dsRNA puncta. MRC-5 cells were infected in triplicate at MOI = 5 with r229E or r229E-nsp15^{mut}. Cells were fixed at 48 hpi and stained for dsRNA using K1 antibody and DAPI. dsRNA puncta were identified using ImageJ, and the number of puncta of each size were quantified. Large puncta were defined as those measuring > 5 μm. Medium puncta were defined as those measuring between 1 and 5 μm

(inclusive). Small puncta were defined as those measuring < 1 μm. The number of foci per field of view in each size bin are shown for each virus, with a linear y-axis in (A) and a $\log_{10}$ scale x-axis in (B). Data shown is from three fields of view quantified in two independent experiments.
